# Caspase-3/Drice as a critical regulator of actin dynamics through its dual control of small RhoGTPase family and Gelsolin in the Malpighian tubules of *Drosophila*

**DOI:** 10.1038/s41420-026-03061-7

**Published:** 2026-04-01

**Authors:** Saurabh Chand Sagar, Madhu G. Tapadia

**Affiliations:** Cytogenetics Laboratory, Department of Zoology, Institute of Science, Banaras Hindu University, Varanasi, India

**Keywords:** RHO signalling, Actin, Morphogenesis

## Abstract

Caspases are well-known executioner enzymes that drive programmed cell death. However, growing evidence indicates their crucial non-apoptotic functions in regulating proliferation, differentiation, endocytic trafficking, cell polarity, morphogenesis, and immune responses. In this study, we uncover a novel role of the *Drosophila* caspase-3 homolog, Drice, in the spatial and dynamic regulation of actin filaments during the development and functional maintenance of Malpighian tubules (MTs). Our previous work demonstrated that Drice is crucial for the morphogenesis of the MTs. Its absence results in erroneous Rho GTPase signaling, driving disarray in actin organization, leading to the formation of multiple fluid-filled cysts in tubules. Here, we show that altered expression of two Rho family GTPases, Rho1 and Cdc42, perturbs downstream signaling in Drice null mutants. Reduced Rok expression aborts Rho1-mediated signaling, whereas elevated Cdc42 levels induce Arp2/3-dependent hyper-polymerization of actin in Drice null mutants. Comparative analyses between control and Drice null mutant MTs revealed loss of the Gelsolin–Rho1 interaction and significant downregulation of Gelsolin expression, disrupting the F-actin: G-actin balance in Drice null mutants. Together, our findings establish a previously unrecognized role of caspase-3/Drice in regulating actin homeostasis and tubule morphogenesis, underscoring its broader significance beyond apoptosis in developmental and physiological contexts.

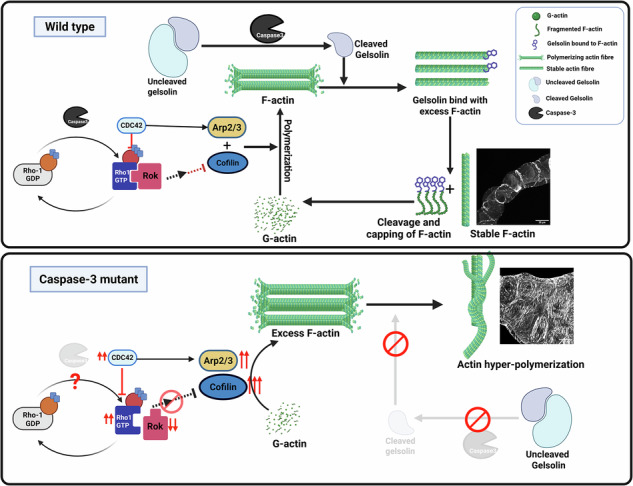

## Introduction

Morphogenesis is a fundamental process in development, as it defines how cells acquire their shape and spatial organization—both of which are indispensable for conventional morphology and organ physiology. This transformation from an undifferentiated cell mass into specialized functional structures relies on the coordinated remodeling of individual cell shapes and positions [1, 2]. A critical element of this remodeling is the close interplay between the plasma membrane and the actin cytoskeleton, which together safeguard cellular integrity while enabling morphological changes and cellular movement/motility [3, 4].

Actin filaments provide the structural framework, and their dynamic switching between monomeric (G-actin) and filamentous (F-actin) supports cell migration, shape transitions, tissue metamorphosis and differentiation [5, 6]. Typically, cytosolic G-actin levels are high, but in response to signaling cues, it polymerizes into F-actin; conversely, F-actin can depolymerize when required by the cell [7, 8]. This dynamic equilibrium is regulated by a diverse set of actin-binding proteins (ABPs), including filament nucleators (such as formins and spire proteins), branching regulators (e.g., Arp2/3 activators), severing factors (such as Gelsolin and villin), and proteins that cap or stabilize filaments [9–13]. The recruitment and activity of ABPs are directed by upstream signaling networks, such as the PI3K-AKT pathway, the Rho family proteins, the Yorkie-Hippo pathway, the MAPK pathway, mTOR, Calcium-dependent regulation, Wnt signaling, etc. Of these, the Rho GTPase family represents a central hub in actin regulation [14, 15]. These molecular switches alternate between active GTP-bound and inactive GDP-bound states, under the control of guanine nucleotide exchange factors (GEFs), GTPase-activating proteins (GAPs), and RhoGDIs [16, 17]. Among the more than 20 known Rho GTPases, RhoA, Rac1, and CDC42 are particularly important in actin regulation [18, 19]. RhoA stimulates stress fibre assembly by activating effector proteins that promote filament bundling [20]. Rho1 is the *Drosophila* orthologue of the mammalian RhoA protein. In contrast, Rac1 drives lamellipodia formation, and CDC42 triggers filopodia extension, largely through Arp2/3-mediated actin branching and elongation [21]. Beyond their individual roles, RhoA, Rac1, and CDC42 engage in reciprocal regulation, creating a dynamic balance that ensures robust yet flexible control of cytoskeletal architecture and morphogenetic progression.

Caspases, activator and executioner, are best known for their key roles in programmed cell death [22]. However, emerging evidence indicates non-apoptotic functions of certain caspases, including caspase-11, have been implicated in cell migration and actin depolymerization [23, 24]. Several studies highlight functional interactions of caspases with signaling molecules that regulate cytoskeletal dynamics directly or indirectly. For example, caspase-3 indirectly influences actin organization by modulating Rho-associated kinase (Rok), a downstream effector of Rho1 GTPase [25, 26]. Caspase-3 also cleaves and activates Gelsolin, an actin-severing and capping protein, thereby altering actin filament turnover [27–31].

Our previous work has shown that Drice (Drosophila caspase-3 homolog), is necessary for the precise development of MTs, which are primarily associated with excretion and osmoregulation, similar to human kidneys [32]. The MTs, despite expressing active caspase-3, do not undergo apoptosis during metamorphosis, unlike most of the other larval tissues, and are carried into the adult stage. Notably, in caspase-3/Drice–deficient flies, MTs show disrupted actin architecture, characterized by abnormally dense F-actin accumulation and multiple fluid-filled cysts, shorter tubule length and functional impairment [32]. This phenotype is accompanied by dysregulated Rho1 GTPase expression, suggesting a link between caspase signaling and Rho1-mediated cytoskeletal organization [32]. However, the exact role of caspases in the modulation of Rho1GTPase*-*mediated cytoskeletal regulation, as well as other underlying reasons for the failure to achieve normal morphological characteristics of MTs, remained a question.

In this study, we provide a comprehensive analysis of how the expression of two members of the RhoGTPase family, Rho1 GTPase and CDC42, is altered in the absence of Drice and how this disruption affects the morphology of MTs in *Drosophila melanogaster*. We show that in the absence of Drice, the downstream effector of Rho1, expression of Rok is reduced, which eventually leads to the tsr/cofilin activation and increase in actin polymerization. On the other hand, increased expression of CDC42 in the MTs results in enhanced Arp2/3 expression, consequently increasing actin branching. In addition, we observed decreased expression of Gelsolin and demonstrated the absence of Gelsolin and Rho1 interaction in Drice mutants. The cumulative effect of these signaling errors causes an imbalance between the F-actin and G-actin ratios. In addition to redefining the cytoskeletal changes caused by caspase-3, this work highlights the broader role of caspases in developmental and physiological processes beyond cell death.

## Result 1: Rok downregulation disrupts actin organization in the MTs of Drice mutant

**Fig. 1:**
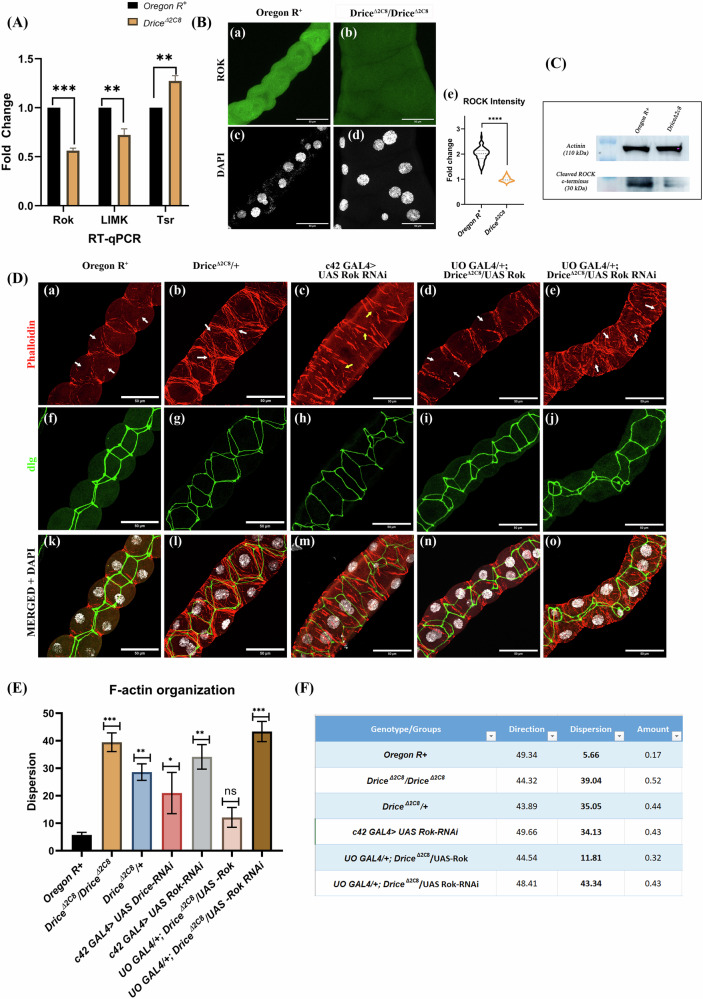
Downregulation of Rok leads to cytosolic actin disruption. **A** Transcript levels of *Rok, LIMK*, and *tsr* in third instar larvae (normalized to *Rp49* as internal control). *Rok* and *LIMK* transcripts are significantly downregulated, whereas *tsr* is significantly upregulated in *Drice*^*Δ2C8*^*/Drice*^*Δ2C8*^ larvae compared to Oregon R^+^. Two-way ANOVA, followed by Tukey’s post hoc multiple-comparison test, was done to determine statistical significance. *p*-value ≤ 0.05 is considered significant, with reference *p < 0.05, ** *p* < 0.01, ***p < 0.001, and ****p < 0.0001. *n* = 3 independent biological replicates. **B** Rok protein expression in Drice mutants: Drice^Δ2C8^/Drice^Δ2C8^ larvae show markedly reduced Rok protein expression (**B**-b, d) compared to Oregon R^+^ (**B**-a, c). Quantification of fluorescence intensity is shown in histogram (**B**-e). Magnitude of scale bar is 50 µm. Statistical significance was determined using a two-tailed Mann–Whitney U test. Data are presented as median with interquartile range from *n* = 3 independent biological replicates. *p*-value ≤ 0.05 is considered significant, with reference *p < 0.05, ** *p* < 0.01, ***p < 0.001, and ****p < 0.0001. Bar graphs are showing Mean ± SEM value. **C** Western blot analysis of Rok protein. Caspase-3 mediated C-terminus cleaved Rok (~30 kDa) is strongly reduced in Drice^Δ2C8^/Drice^Δ2C8^ larvae compared to Oregon *R*^*+*^. Actinin (~110 kDa) was used as a loading control due to variability in actin and tubulin levels between samples [71]. **D** F-actin organization (in red) and apico-basal polarity (green) in the MTs: Oregon R^+^ MTs display continuous cortical F-actin fibres with clear cytosolic regions (**D**-a, f, k; white arrows). In contrast, Drice^Δ2C8^ mutant larvae exhibit dense and disorganized/condensed cytosolic F-actin accumulation with disruption of apico-basal polarity (**D**-b, g, l; white arrows). Knockdown of Rok *(c42-Gal4* > *UAS-Rok-RNAi*) results in thick, discontinuous F-actin fibres (**D**-c, h, m; yellow arrows). Overexpression of Rok in the Drice mutant background (*UO-Gal4/+; Drice*^*Δ2C8*^*/UAS-Rok)* restores F-actin localization and dlg expression (**D**-d, i, n) similar to Oregon R^+^, whereas Rok knockdown in the Drice mutant background (*UO-Gal4/+; Drice*^*Δ2C8*^*/UAS-Rok-RNAi*) further exacerbates F-actin disorganization (**D**-e, j, o). Magnitude of scale bar is 50 µm. **E** Quantitative analysis of actin filament organization. Fourier-based directionality analysis showed low angular dispersion in Oregon R+ tubules, indicative of highly aligned actin filaments. In contrast, Drice mutants (in heterozygous as well as homozygous conditions), Drice-RNAi, and Rok-RNAi increased dispersion, reflecting severe actin disorganization. Notably, Rok overexpression in the Drice mutant background partially restored actin alignment, whereas combined Drice loss and Rok knockdown failed to rescue the phenotype. **F** Tabular representation of the Fourier-based directionality analysis. Welch’s one-way ANOVA test with Tukey’s post hoc test was done to determine the statistical significance. *p*-value < 0.05 is considered significant, with reference *p < 0.05, ** *p* < 0.01, ***p < 0.001, and ****p < 0.0001. Bar graphs are showing Mean ± SEM value. *n* = 5 (technical replicate) * three independent biological replicates.

In our previous study, we reported that a homozygous mutation in Drice, *w*^*1118*^*;+/+; Drice*^*Δ2C8*^*/Drice*^*Δ2C8*^, results in abnormal development of the MTs. They exhibit distorted morphology along their length with numerous fluid-filled cysts [32]. Drice mutant’s MTs develop extensive accumulation of actin filaments and enhanced Rho1GTPase expression (Supplementary Fig. S1) as reported earlier [32]. The question we are addressing now is whether actin dynamics is modulated solely by the disruption of Rho1GTPase, or if other members of the Rho family, *viz*., Rac and CDC42, are also perturbed in the absence of Drice. We first examined the expression of Rok, LIMK and Tsr, the immediate downstream effector molecules of Rho1GTPase, by qRT-PCR in the MTs from 3rd instar larvae. Transcript analysis revealed a significant reduction in mRNA levels of Rok and LIMK, along with an upregulation in the mRNA levels of Tsr in Drice^Δ2C8^/Drice^Δ2C8^ larvae compared with Oregon R^+^ (Fig. 1A). In order to find out whether reduced mRNA levels also resulted in reduced protein levels, immunostaining was performed with anti-Rok antibody. A distinct reduction was visible in the MTs of Drice^Δ2C8^/Drice^Δ2C8^ (Fig. 1B-b, d) compared with Oregon R^+^ (Fig. 1B-a, c). Rok remains in a self-inhibitory state under basal conditions, and is activated either through Rho1 association at the Rho-binding domain or via proteolytic cleavage of the C-terminal cysteine-rich region by caspase-3 [25, 33, 34]. Western blot analysis using an anti-Rok antibody further confirmed a significant reduction in Rok protein levels in *Drice*^Δ2C8^/*Drice*^Δ2C8^ larvae (Fig. 1C) (full blot attached in Supplementary Fig. S3). To further understand the role of Rok in the MTs, it was downregulated by employing the UAS-Gal4 system [35]. MT specific Gal4, *c42*, was used to express *UAS-Rok RNAi*. It was observed that in the MTs of *c42-Gal4* > *UAS-Rok RNAi* progeny, F-actin filaments appeared to be thicker and the regular arrangement was lost (Fig. 1D-c, m; yellow arrows) as compared to Oregon R^+^ (Fig. 1D–a, k), but not to the extent of Drice mutants (Fig. 1D–b, l). Additionally, Rok knockdown, viz*., c42-Gal4* > *UAS-Rok RNAi*, also resulted in extra budding and morphological defects in the tubules (Supplementary Fig. S2), indicating the role of Rok in tissue morphogenesis. Having observed that knockdown of Rok disrupted actin organization, we overexpressed Rok in the Drice mutant background using MT specific *UO-Gal4*. As expected in the MTs of *UO-Gal4/+; Drice*^*Δ2C8*^*/UAS-Rok*, progeny, a significant reduction of cytosolic F-actin accumulation along with restoration of F-actin organization resembling Oregon R^+^ was observed (Fig. 1D-d, n), when compared with Drice mutants either in heterozygous (Fig. 1D–b, l) or homozygous condition (Supplementary Fig. S7A-p, r). The morphology of the tubules also reverted to a similar one to Oregon R^+^ (Fig. 1D-a, k). On the contrary, Rok knockdown in the Drice mutant background (*UO-Gal4/+; DriceΔ2c8/UAS-Rok-RNAi*) led to further disruption of F-actin in the MTs (Fig. 1D-e, o). We checked the actin-organization and disorganization by the Fourier-based directionality analysis (detailed description is available in the method section), which revealed marked disruption of actin filament organization upon loss of Drice and Rok activity. Control Oregon R+ tubules exhibited low angular dispersion values (5.66°), consistent with highly aligned actin filaments. In contrast, both homozygous and heterozygous Drice^Δ2C8^ mutants showed a pronounced increase in dispersion (39.04° and 35.05°, respectively), indicating severe actin disorganization. Similarly, *Rok-RNAi* phenocopied the Drice mutant condition, resulting in high dispersion values (34.13°). Notably, overexpression of Rok in the Drice mutant background substantially reduced angular dispersion (11.81°), reflecting partial restoration of actin organization. Conversely, combined Drice loss and Rok knockdown failed to rescue actin alignment and instead maintained high dispersion values (43.34°) (Fig. 1E, F and Supplementary Fig. S8).

Disc large (Dlg) is a junctional protein that marks the basolateral membrane and is essential for maintaining apico–basal polarity of the tubules [36, 37]. In Drice mutants, Dlg expression was severely disrupted (Fig. 1D–g), reflecting disruption in the morphology compared with wild-type (Fig. 1D–f). Similar to Drice, Dlg disruption was observed in *Rok-RNAi* (Fig. 1D–h); however, this defect was rescued in *UO-Gal4/+; Drice*^*Δ2c8*^*/UAS-Rok* progeny (Fig. 1D–i). Taken together, these findings suggest that the high cytosolic F-actin accumulation in Drice mutant MTs results from an imbalance in the downstream effectors of the Rho1 pathway. These findings suggest a potential role for Rok in regulating the morphogenesis of the MTs.

## Result 2: Overexpression of CDC42 causes disarrayed actin organization in the MTs

**Fig. 2:**
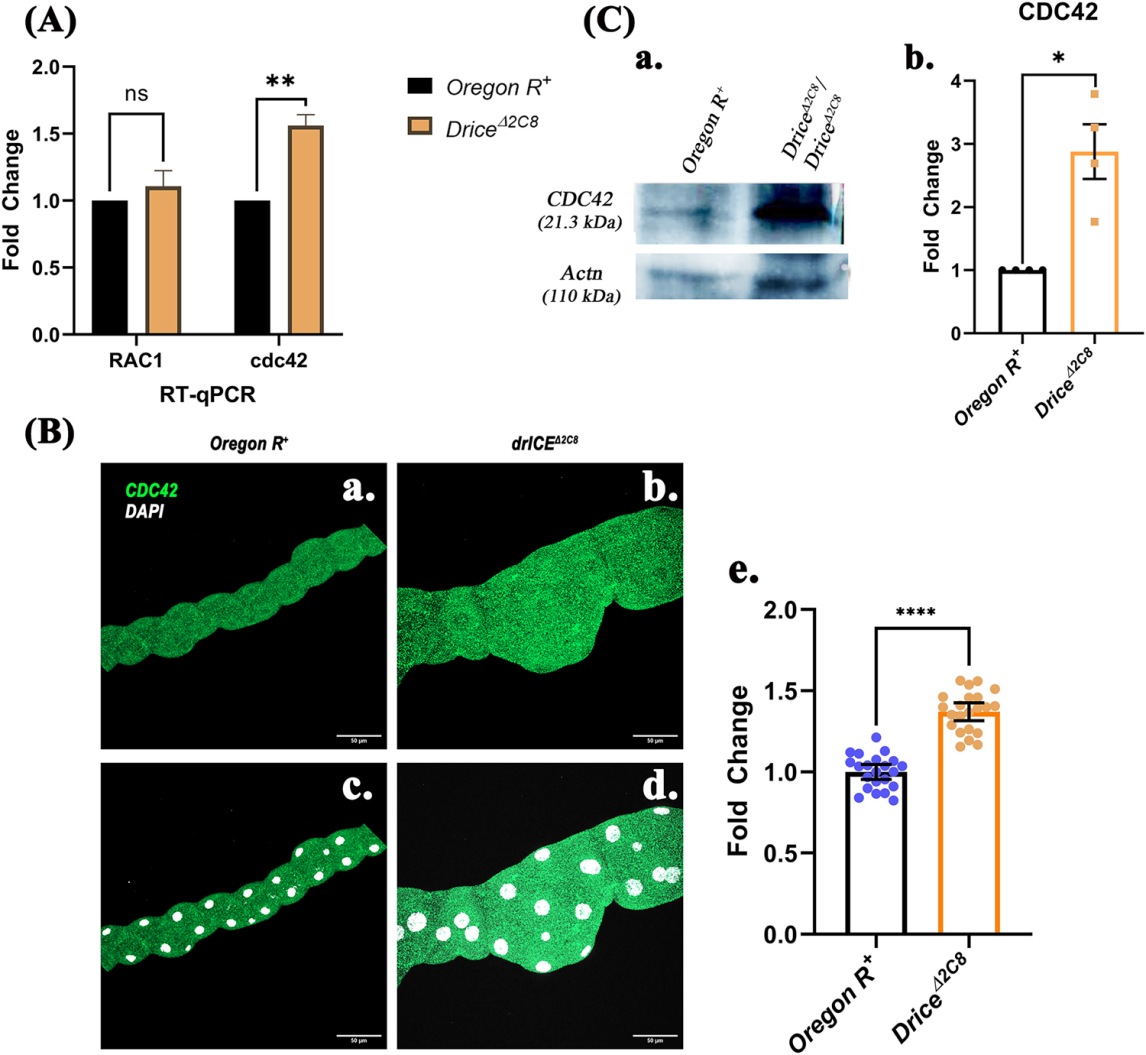
Upregulation of transcript and protein levels of CDC42 in Drice mutants. **A** Transcript levels of the Rac and CDC42 in the MTs of 3rd instar *Drice*^*Δ2C8*^*/Drice*^*Δ2C8*^ larvae when compared to the Oregon R^+^ in qRT-PCR (RP49 was used as internal control). Two-way ANOVA, followed by Tukey’s post hoc multiple-comparison test, was done to determine statistical significance. *p*-value ≤ 0.05 is considered significant, with reference *p < 0.05, ** *p* < 0.01, ***p < 0.001, and ****p < 0.0001. *n* = 3 independent biological replicates. **B** Elevation of CDC42 in the immunostaining: CDC42 protein is significantly upregulated in the MTs of wandering 3rd instar larvae of DriceΔ2C8/DriceΔ2C8 (**B**-b, d) and in Oregon R+ (**B**-a, c) and their quantification (**B**-e). Scale bar is 50 µm). Statistical significance was determined using a two-tailed Mann–Whitney U test. *p*-value ≤ 0.05 is considered significant, with reference *p < 0.05, ** *p* < 0.01, ***p < 0.001, and ****p < 0.0001. Bar graphs are showing Mean ± SEM value. *n* = 5 (technical replicate) * three independent biological replicates. **C** Western blot analysis of the CDC42 protein in DriceΔ2C8/DriceΔ2C8 and Oregon R+ (Actinin was used as internal control antibody). Statistical significance was determined using a two-tailed Mann–Whitney U test. *p*-value ≤ 0.05 is considered significant, with reference *p < 0.05, ** *p* < 0.01, ***p < 0.001, and ****p < 0.0001. Bar graphs are showing Mean ± SEM value. Data are presented as median with interquartile range from *n* = 3 independent biological replicates.

Given the dysregulation of the Rho1 downstream cascade, we next examined the status of other Rho GTPase family members, namely Rac and CDC42. The interaction between Rho1 and Rac is known to be context dependent, leading to either mutual activation or antagonism depending on the dominant Rho1 target within the cell [38]. On the contrary, CDC42 has been identified as a key inhibitor of Rho1 activation during cytokinesis in early anaphase [39]. qRT-PCR was done to check the status of transcription of *Rac* and *CDC42* in the MTs of 3rd instar larvae. Transcript analysis did not reveal a significant change in Rac expression, whereas CDC42 was significantly upregulated in *Drice*^*Δ2C8*^*/Drice*^*Δ2C8*^ larvae (Fig. 2A). Based on these observations, we further analyzed the role of CDC42. Immunostaining of 3rd instar larval MTs with anti-CDC42 antibody showed a significant increase in CDC42 expression in *Drice*^*Δ2C8*^*/Drice*^*Δ2C8*^ larvae (Fig. 2B- b, d) compared with Oregon R^+^ (Fig. 2B- a, c) upon intensity-based quantification (Fig. 2B-e). Western blot analysis further confirmed the enhanced expression of the CDC42 (Fig. 2C), suggesting that an increase in CDC42 beyond a critical level led to disruption in the development of MTs.

## Result 3: Arp-mediated over-accumulation of F-actin in Drice mutants

**Fig. 3:**
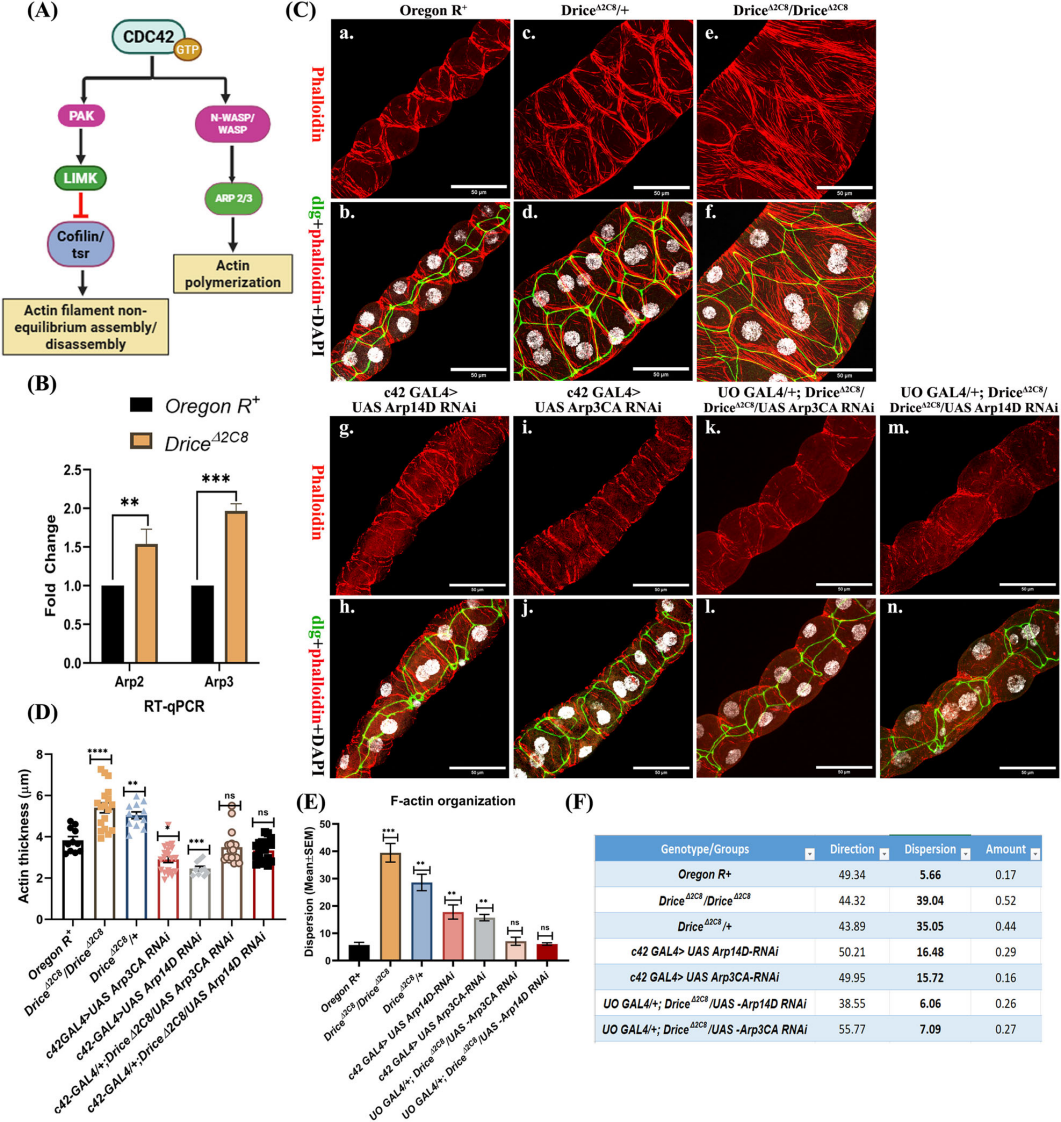
Role of Arp in the actin branching and polymerization. **A** Downstream effectors of the CDC42. **B** Transcript levels of the Arp2 & Arp3 in the MTs of 3^rd^ instar *Drice*^*Δ2C8*^*/Drice*^*Δ2C8*^ larvae when compared to the Oregon R^+^ in qRT-PCR (RP49 was used as internal control). Two-way ANOVA, followed by Tukey’s post hoc multiple-comparison test, was done to determine statistical significance. *p*-value ≤ 0.05 is considered significant, with reference *p < 0.05, ** *p* < 0.01, ***p < 0.001, and ****p < 0.0001. *n* = 3 independent biological replicates. Bar graphs are showing Mean ± SEM value. **C** Arp knockdown led to the thinning of the cortical F-actin filaments: Dense actin fibres at the cell cortex in Drice mutants (homozygous, **C**-e, f; heterozygous, **C**-c, d) when compared to the Oregon R^+^ actin localization within the MTs (**C**-a, b). Knockdown of Arp2 (*c42-Gal4>UAS Arp14D RNAi*) (**C**- g, h) led to the incomplete and thinner actin filaments at the cell cortex; also, knockdown of Arp3 (*c42-Gal4>UAS Arp3CA RNAi*) (**C**-i, j) led to the thinner actin filaments at the cell cortex in the MTs. Further, Arp3 knockdown in Drice mutant background (**C**- k, l) and Arp2 knockdown in Drice mutant background (**C**–m, n), restores the actin thickness at the cell cortex. Figure a-l showing 40×1.0 zoom image; red, green and gray are showing phalloidin, dlg and DAPI, respectively (scale bar = 50 µm). **D** F-actin thickness in different genetic backgrounds. Actin filaments are thickened in the Drice mutant background when compared to the wild type. Arp2 and Arp3 knockdown led to the tinning of the cortical F-actin when compared to the wild type. Arp2 and Arp3 knockdown in the Drice mutant background restored the F-actin filament thickness to normal. Welch’s one-way ANOVA test with Tukey’s post hoc test was done to determine the statistical significance. *p*-value < 0.05 is considered significant, with reference *p < 0.05, ** *p* < 0.01, ***p < 0.001, and ****p < 0.0001. Bar graphs are showing Mean ± SEM value. *n* = 5 (technical replicate) * three independent biological replicates. **E** Quantitative analysis of actin filament organization. Fourier-based directionality analysis showed low angular dispersion in Oregon R+ tubules, indicative of highly aligned actin filaments. In contrast, Drice mutants (in heterozygous as well as homozygous conditions), Arp14D-RNAi, and Arp3CA-RNAi increased dispersion, reflecting severe actin disorganization. Notably, Arp14D-RNAi and Arp3CA-RNAi overexpression in the Drice mutant background partially restored actin alignment. **F** Tabular representation of the Fourier-based directionality analysis. Welch’s one-way ANOVA test with Tukey’s post hoc test was done to determine the statistical significance. *p*-value < 0.05 is considered significant, with reference *p < 0.05, ** *p* < 0.01, ***p < 0.001, and ****p < 0.0001. Bar graphs are showing Mean ± SEM value. *n* = 5 (technical replicate) * three independent biological replicates.

Having observed upregulation of CDC42, we next investigated the downstream effectors of CDC42 in Drice mutants. CDC42 interacts with WASP to activate it, which subsequently recruits the Arp2*/*3 complex, promoting nucleation and branching of actin filaments (Fig. 3A) [40–42].

Transcript levels of both Arp2 and Arp3 were significantly upregulated in *Drice*^*Δ2C8*^*/Drice*^*Δ2C8*^ mutants, as shown by qRT-PCR (Fig. 3B). To assess functional relevance, we downregulated Arp2 by driving *UAS Arp14D-RNAi* with *c42-Gal4*. In *c42Gal4* > *UAS-Arp14D-RNAi* progeny (Fig. 3C- g, h), the actin filaments appeared to be disrupted, thin and discontinuous in MTs as observed in the Drice mutant background (Fig. 3C-c-f). Similarly, actin filaments in knockdown of *Arp3, viz., c42-Gal4* > *UAS-Arp3CA-RNAi* progeny, were comparable to those of *Arp2 RNAi* (Fig. 3C-i, j). Further, the Arp2 knockdown in Drice mutant background (Fig. 3C-m, n), and Arp3 knockdown in Drice mutant background (Fig. 3C-k, l) restored the actin filament thickness back to the normal as wild type (Fig. 3C-a, b). Quantification of the actin filament thickness (detailed description available in methods sections) revealed that actin at the cell cortex in wild type was 3.64 µm which was increased significantly to the 5.40 µm in Drice homozygous and 5.08 µm in Drice heterozygous mutants. Similarly, actin filament thickness was reduced to 2.90 µm and 2.44 µm in Arp2 and Arp3 knockdown background, respectively (Fig. 3D). Arp2 and Arp3 knockdown in the Drice mutant background restores the actin thickness to 3.49 µm & 3.33 µm, respectively, closely resembling the wild type phenotype (Fig. 3D). Similar to the result 1, Fourier-based directionality analysis for actin organization revealed that perturbation of the Arp2/3 complex using c42-GAL4–driven RNAi against Arp14D or Arp3CA resulted in moderate increases in angular dispersion (16.48° and 15.72°, respectively), indicating partial disorganization of actin filaments relative to controls. Importantly, suppression of Arp2/3 activity in the Drice mutant background markedly reduced angular dispersion to near-control levels (6.06° for Arp14D RNAi and 7.09° for Arp3CA RNAi), reflecting substantial restoration of actin alignment. These quantitative data suggest that excessive Arp2/3-mediated actin branching contributes to the disorganized actin architecture observed in Drice-deficient tubules and that limiting Arp2/3 activity can effectively rescue actin filament organization in this context (Fig. 3E, F and Supplementary Fig. S8).

Since the Arp2/3 complex creates branching of the actin filaments, it can be hypothesized that their enhanced expression could be responsible for excess networking of actin fibres in the Drice mutants (Fig. 3D- c-f) and reduction in Arp by RNAi (Fig. 3D- g-j) reduces branching. These observations provide strong evidence that the Arp2/3 complex has a critical role in the regulation of cortical actin in MTs.

## Result 4: Gelsolin, a mediator between Drice and Rho1GTPase, in the regulation of the actin dynamics within the MTs

After exploring the roles of Rho1-GTPase and CDC42 in actin dynamics in MTs, a critical question remained: What role does *Caspase-3/Drice* play in coordination with Rho1 family members in regulating the actin cytoskeleton? To address this, immunoprecipitation-mass spectrometry (IP-MS) was performed from the larval MTs lysate of Oregon R^+^ and Drice mutants using an anti-Rho1 antibody to identify the interacting partners of Rho protein in the different genetic backgrounds. A total of 235 interactors in Oregon R^+^ and 264 interactors in Drice mutant were identified interacting with Rho1. Of these, 211 interactors were common in both the genotypes, while 24 interactors were unique in Oregon R^+^, and 53 in Drice mutants. Several proteins like troponin, flare, cpa, cpb, Arp3, and tropomyosin-I were unique interactors of Rho1 in Drice mutants, whereas proteins like Gelsolin, myosin, moesin, cora, lva etc. were unique interactors identified in Oregon R^+^ (Supplementary Table S1). Among these, Gelsolin was of particular interest, as it severs and caps F-actin and is a substrate of the Caspas-3 as well.

**Fig. 4:**
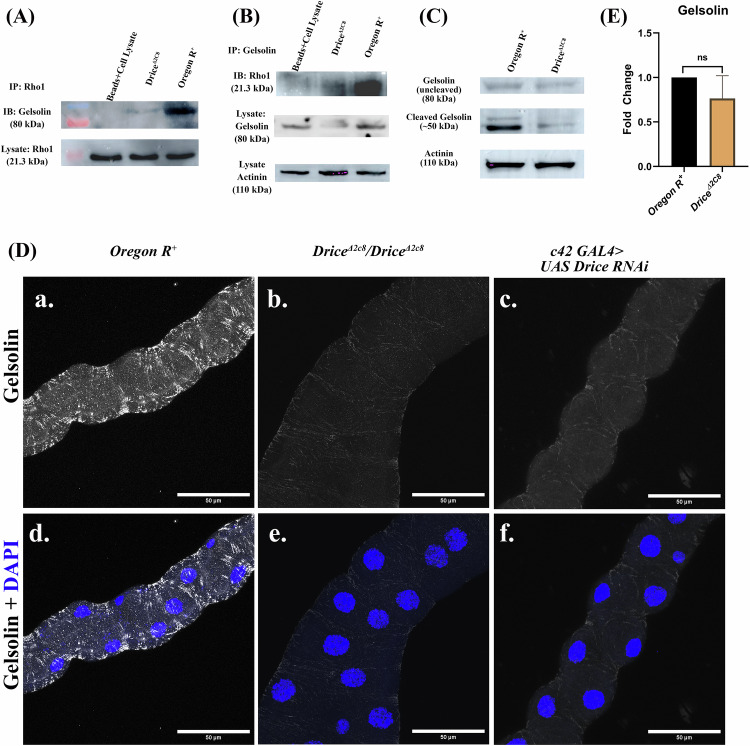
Gelsolin activity in Oregon R^+^ and Drice mutants. Co-immunoprecipitation (Co-IP) from whole-body lysates of 118–120 h third instar wandering stage larvae using anti-Rho1 or anti-Gelsolin antibodies. **A** Rho1-immunoprecipitated lysate probed with anti-Gelsolin antibody showed no detectable interaction in *Drice*^*Δ2C8*^/*Drice*^*Δ2C8*^ mutants, whereas the interaction was present in Oregon R^+^. **B** Gelsolin-immunoprecipitated lysates from *Drice*^*Δ2C8*^/*Drice*^*Δ2C8*^ mutants showed no interaction with Rho1. In contrast, both Rho1–Gelsolin and Rok–Gelsolin interactions were present in Oregon R^+^. **C** Western blot analysis of Gelsolin protein using anti-Gelsolin antibody. Cleaved Gelsolin (*~*50 kDa) was significantly reduced in *Drice*^*Δ2C8*^/*Drice*^*Δ2C8*^ mutants compared with Oregon R^+^. A baseline level of Uncleaved Gelsolin (*~*80 kDa) was detected in both genotypes. Actinin (*~*110 kDa) was used as a loading control. **D** Immunostaining of MTs using anti-Gelsolin antibody (gray). Gelsolin levels were markedly reduced in *Drice*^*Δ2C8*^/*Drice*^*Δ2C8*^ mutants (**D**-b, e) compared with Oregon R^+^ (**D**-a, d). Knockdown of Drice (*c42-Gal4* *>* *UAS-Drice RNAi*) showed a similar reduction, consistent with Drice mutants. The magnitude of the scale bar is 50 µm. **E** Transcript levels of the Gelsolin in the MTs of 3^rd^ instar *Drice*^*Δ2C8*^/*Drice*^*Δ2C8*^ larvae when compared to the Oregon R^+^ in qRT-PCR (RP49 was used as internal control). Statistical significance was determined using a two-tailed Mann–Whitney U test. *p*-value ≤ 0.05 is considered significant, with reference *p < 0.05, ** *p* < 0.01, ***p < 0.001, and ****p < 0.0001. Bar graphs are showing Mean ± SEM value. Data are presented as median with interquartile range from *n* = 3 independent biological replicates.

To assess potential interactions between Rho1 and Gelsolin, we performed co-immunoprecipitation (co-IP) experiments. Immunoprecipitation using an anti-Rho1 antibody followed by immunoblotting with an anti-Gelsolin antibody revealed minimal to undetectable association between Rho1 and Gelsolin in Drice^Δ2C8^/Drice^Δ2C8^ mutants (Fig. 4A; full blot, Supplementary Fig. S4). This result was verified by reversing the experimental conditions—immunoprecipitating with an anti-Gelsolin antibody and probing with an anti-Rho1 antibody. Comparatively low interaction was observed between Gelsolin and Rho proteins in Drice mutants when compared with Oregon R^+^, reflecting the similar trend seen earlier (Fig. 4B; full blot Supplementary Fig. S5).

Gelsolin is an ~80 kDa actin-severing protein that requires Caspase-3–mediated cleavage for activation [27, 43]. Upon cleavage, the N-terminal fragment severs and caps actin filaments at the barbed end, thereby preventing further actin polymerization [28–31]. Following the IP-MS data, we did a western blot to further validate Gelsolin interaction, which revealed a significant reduction in the cleaved Gelsolin in the Drice mutants when compared to Oregon R^+^ (Fig. 4C; full blot in Supplementary Fig. S6). Further, we checked the level of Gelsolin protein by immunostaining in Drice mutants and in Oregon R^+^, and a significant reduction of Gelsolin expression in Drice mutants (Fig. 4D-b, e) was observed when compared with *Oregon R*^*+*^ (Fig. 4D-a, d). *c42 Gal4* > *UAS Drice RNAi* MTs exhibited a similar reduction in Gelsolin protein expression (Fig. 4D-c, f) as in Drice mutants, further supporting the requirement of Drice for Gelsolin expression. However, at the transcript level non-significant difference was observed in Drice mutants as compared to wild type.

Together, these findings suggest that Caspase-3/Drice activity is required for Gelsolin expression, and thus failure in interaction with Rho1. The dense actin filaments observed in *Drice*^*Δ2C8*^*/Drice*^*Δ2C8*^ mutants could be a result of the absence of Gelsolin-mediated actin severing activity.

## Result 5: Caspase-dependent regulation of F-actin: G-actin ratio within the MTs

**Fig. 5:**
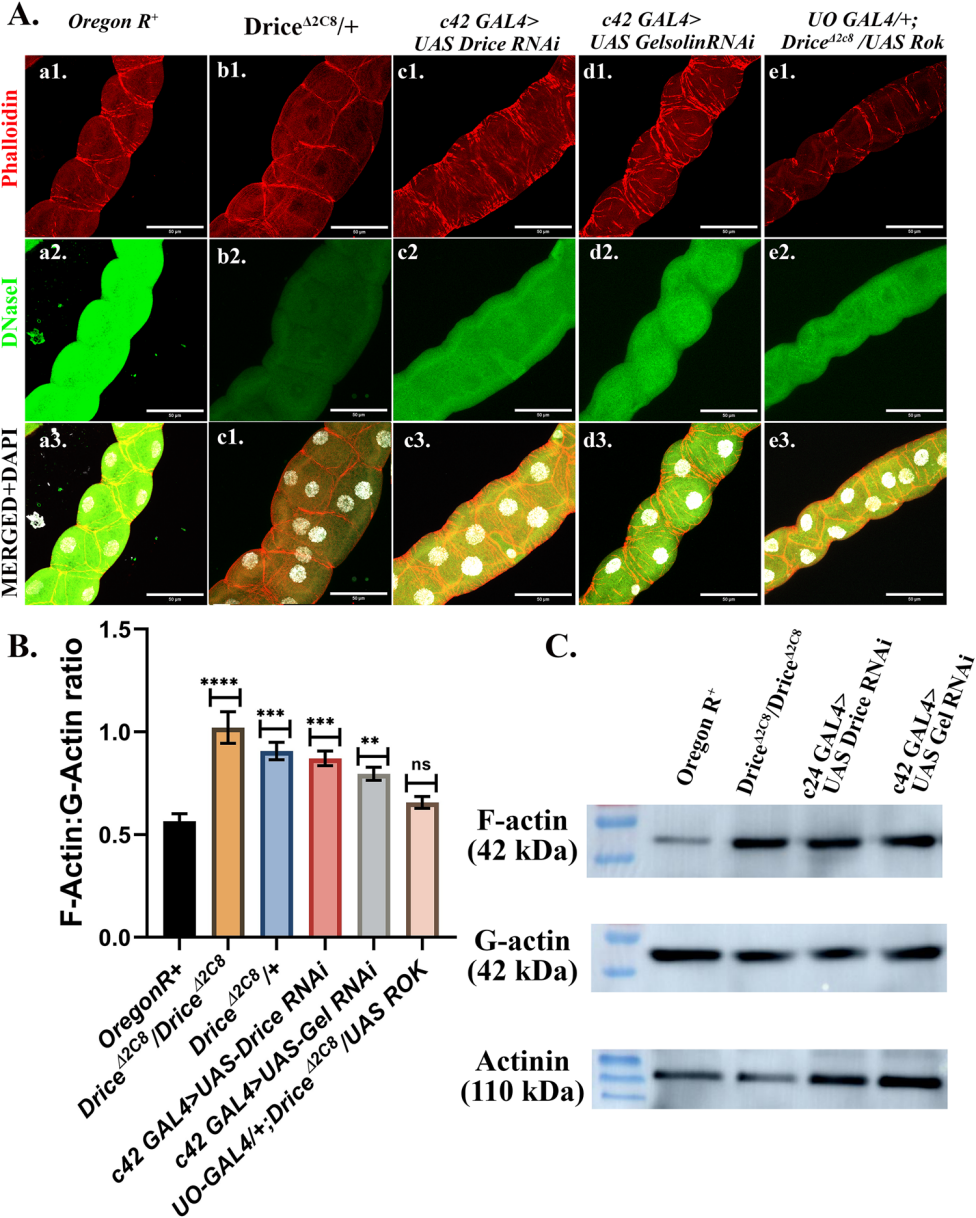
F-actin vs. G-actin dynamics in MTs. **A** Confocal projection images of MTs from wandering third instar larvae (118–120 h) showing F-actin (Phalloidin, red), G-actin (DNaseI, green), and nuclei (DAPI, gray) under different genetic backgrounds. F-actin and G-actin levels in Oregon R^+^ (a1-a3); disorganization in F-actin (organization and level) and reduced G-actin levels in Drice knockdown viz., Drice mutants (b1-b3), *Drice-RNAi* (c1-c3), and *Gelsolin RNAi* (d1-d3) can be seen; while Rok overexpression in Drice mutant background *viz., UO-Gal4/+; Drice*^*∆2C8*^*/UAS-Rok* (e1-e3) increases G-actin level with and improvement in f-actin organization similar as in Oregon R^+^. The magnitude of the scale bar is 50 µm. **B** Quantification of F-actin and G-actin proportions across various genetic backgrounds*:* *F-/G- actin r*atio is elevated in all *Drice-knockdown* backgrounds (Drice mutants, *Drice RNAi)* and in Gelsolin knockdown (*Gelsolin-RNAi*). Rok overexpression in the Drice mutant background (*UO-Gal4/+*; *Drice*^*∆2C8*^*/UAS-Rok*) rescues the ratio to wild-type levels. Welch’s one-way ANOVA test with Tukey’s multiple comparison test was done to determine the statistical significance. *p*-value < 0.05 is considered significant, with reference *p < 0.05, ** *p* < 0.01, ***p < 0.001, and ****p < 0.0001. Bar graphs are showing Mean ± SEM value. *n* = 5 (technical replicate) * three independent biological replicates. **C** Western blot analysis of the F-actin and G-actin levels. Elevated F-actin levels can be seen in the Drice and Gelsolin knockdown background. Also, a reduction in G-actin levels is evident in the same genetic background. This further supports the immunostaining findings.

Transitioning between G-actin to F-actin is critical for cell movement-related processes. Having observed severe impairment of several proteins involved in actin assembly/disassembly, we wanted to check if it correlated with the F-actin to G-actin ratio. To address this question, we quantified the relative abundance of F- and G- actin under different genetic backgrounds (Fig. 5B). F-actin was quantified by staining with phalloidin, and G-actin was stained using DNase I dye. The ratio was calculated by quantifying the fluorescence intensity. Reduced expression of G-actin was observed in *Drice*^*Δ2C8*^*/+* (Fig. 5A-b2) and *c42GAL4* > *UAS-Drice RNAi* (Fig. 5A-c2) as compared to Oregon R^*+*^ (Fig. 5A-a2) and driver *c42* alone (Supplementary Fig. S7), but an increase in the F-actin levels in the Drice knockdown background (Fig. 5A-b1, c1). Further, *Rok-RNAi, Arp3CA-RNAi* and *Arp14D-RNAi* (Supplementary Fig. S7) do not significantly affect either F-actin or G-actin expression levels; however, they affect the F-actin organization as discussed earlier (result 3). In Gelsolin knockdown (Fig. 5A-d1-d3), however, the G-actin was significantly reduced with relatively no change in the expression levels of the F-actin. Further, in *UO-Gal4/+; Drice*^*∆2C8*^*/UAS-Rok* (Fig. 5A-e1-e3), G-actin levels increased significantly when compared to the Drice mutant, though it was not the same as wild type (Fig. 5A-e2; and Supplementary Fig. S7D).

Having observed this trend, we examined the F-actin: G-actin Ratio in the other genotypes (*Rok-RNAi, Arp3CA RNAi, Arp14D RNAi)*, which affected actin organization. In Oregon R^+^, the F-actin: G-actin ratio in the MTs was ~0.56 (Fig. 5B), consistent with a balanced state of actin turnover in the absence of active polymerization or depolymerization [44, 45]. In contrast, Drice mutants, both in homozygous as well as heterozygous condition exhibited a marked elevation in the ratio of F-/G-actin ~1.00, directly correlating with enhanced actin fibres. A similar trend was observed in the F-actin: G-actin ratio in *c42Gal4* > *UAS-Drice RNAi* (~0.85). Following the above results, we next sought to look at F-actin: G-actin ratio in *c42Gal4* > *UAS-Gelsolin RNAi*, which was ~0.80, suggesting enhanced actin polymerization (Fig. 5B). Similarly, knockdown of *Rok, Arp3CA*, or *Arp14D* did not result in any deviation from Oregon R^*+*^ F-/G- actin ratio, *viz., c42Gal4* > *UAS RokRNAi* ~ *0.58, c2Gal4* > *UAS Arp3CA-RNAi* ~ *0.582, and c42 Gal4* > *UAS Arp14D-RNAi* ~ *0.67*; suggesting these effectors may not be the primary determinants of actin remodeling in the MTs (Supplementary Fig. S7, C).

A significant observation was that overexpression of Rok in the Drice mutant background (Fig. 5A, e1-e3) restored the F-actin: G-actin ratio to ~0.60, similar to the Oregon R^+^, suggesting that Rok activation is specifically impaired in the absence of caspase activity. Based upon these observations, it can be speculated that Drice and Gelsolin are key regulators of actin dynamics in the MTs by regulating the actin polymerization/depolymerization state; Rok, Arp2 and Arp3, however, appear to regulate the actin organization rather than affecting the actin steady state dynamics directly.

## Discussion

Microfilaments are major cytoskeletal components steering tissue architecture. The crucial balance between assembly and disassembly of actin filaments is essential for coordinated cell movement, necessary for precise positioning and hence proper physiological functions [5, 6]. It regulates morphogenetic processes by providing a scaffold that allows cell adhesion and cell migration, thereby enabling the formation of three-dimensional structures during development and organogenesis [46–48]. Several signaling pathways, including small GTPases, PI3K, Hippo, and mTOR, regulate actin dynamics within cells [49, 50]. Among these, small GTPase family proteins are key regulators of actin organization, and thus critical determinants of proper cell orientation, movement, morphology, and function [19]. Caspases, on the other hand, are traditionally associated with apoptosis and cell death; however, their non-apoptotic roles have recently emerged as an important area of research [32, 51–54]. In the present study, we report that altered expression of Rho1 and CDC42 have profound effect on the development of MTs, which has also been observed during murine kidney tubule development [55].

Although Rho1 levels were high in the MTs, it probably failed to relay the signal to the downstream effector, Rok, as Rok was downregulated at transcriptional and subsequent translational levels. Rok has an inhibitory effect on cofilin/twinstar, the actin-severing actin protein [56]. In the absence of Rok, this inhibition is lifted in Drice homozygous mutants, resulting in increased polymerization of F-actin. The other member of the Rho family, CDC42, through WASP family proteins [57], activates the Arp2/3 complex. This complex associates with pre-existing filaments to initiate new filaments, by actin nucleation, uncapping, or severing filaments [58, 59]. Enhanced expression of CDC42 in Drice mutants could be responsible for enhanced expression of the Arp2/3 complex, which correlates with dense cortical actin fibres [60–62]. Conversely, knockdown of Arp2/3 led to thinning of actin fibres, implying that the actin hyper-polymerization observed in Drice mutants is mediated through the Arp2/3 complex.

Rho-Gelsolin interaction has not been reported earlier, which suggests a novel and tissue-specific observation. Gelsolin, being an actin-severing and capping protein, is regulated either by caspase-mediated cleavage or by calcium signaling [27, 28, 31, 63, 64]. In the absence of Caspase-3, Gelsolin probably remains in its inactive form, failing to sever and cap excess F-actin, thereby exacerbating cytoskeletal imbalances. Thus, Gelsolin could represent a crucial target of Drice that functions in maintaining appropriate F-actin turnover.

The balance between the dynamic states of G-actin and F-actin reflects the physiological status of actin turnover within cells [65]. In steady-state conditions, the proportion of G-actin typically exceeds F-actin, with reported F: G ratios ranging from ~0.66 to ~0.33, indicative of basal equilibrium without active remodeling [44, 45, 66, 67]. In contrast, cells undergoing migration display a shift toward higher F-actin abundance relative to G-actin, consistent with active filament assembly [44, 68]. The shift in the G- and F-actin pools observed in the absence of Drice could be driving excessive polymerization, conforming with an earlier study suggesting this ratio determines the fate of actin polymerization in the biological system [69]. Under steady-state conditions without active polymerization, G-actin is higher than F-actin, whereas high F-actin content represents an active polymerization state [44, 45, 66, 67]. In accordance with this, a high F-actin:G-actin ratio was reported in Drice mutants and Gelsolin knockdown background, implicating active actin polymerization in these two. Further quantification revealed that F-actin levels remained largely unaltered across *Rok RNAi, Arp3CA-RNAi, Arp14D-RNAi* backgrounds, suggesting the regulation of actin organization without altering the actin steady state. Importantly, overexpression of Rok in the Drice mutant background normalized the F-actin: G-actin ratio by improving the G-actin levels, indicating Rok activity can partially rescue cytoskeletal defects in the absence of caspase activity. This rescue was absent under Rok knockdown, highlighting a unique Rok-dependent regulatory mechanism in Drice mutants. As G-actin is the building block for the formation of F-actin [70], and the level of G-actin is very low in Drice and Gelsolin knockdown backgrounds, suggesting that reduced G-actin in these genotypes could be a result of excessive polymerization into the F-actin. When Rok is overexpressed in the Drice mutant background, it partially rescues the F-/G- actin ratio in the MTs. Since Gelsolin is severing and capping F-actin, in its absence, the severing of F-actin in Drice mutants is reduced, further disrupting the F-actin organization. The failure to trigger Rho1 downstream effectors despite high Rho1 protein could be due to the absence of Drice. However, the exact mechanistic regulation of the Rho1 in a Caspase-depleted background is still elusive and yet to be fully understood.

Compelling data presented in this study suggest that Drice could be crucial in coordinating Rho family signaling and activity to organize the actin filaments correctly in MTs. The results highlight Caspase-3/Drice as a central regulator of actin dynamics through its dual control of Rok and Gelsolin. The interplay between caspase activity and actin regulators ensures a proper balance between F-actin and G-actin pools, which in turn influences MTs morphology. Our study indicates that the absence of Drice could be altering the function of several actin assembly proteins and could significantly affect the convergent extension movement necessary for MTs morphogenesis. These findings broaden the functional repertoire of caspases beyond apoptosis, positioning them as critical modulators of cytoskeletal dynamics. Future studies focusing on the precise regulation of Rho1 activity in caspase-deficient backgrounds will be instrumental in fully understanding the crosstalk between caspase signaling, actin remodeling, and microtubule organization.

## Summary

**Fig. 6:**
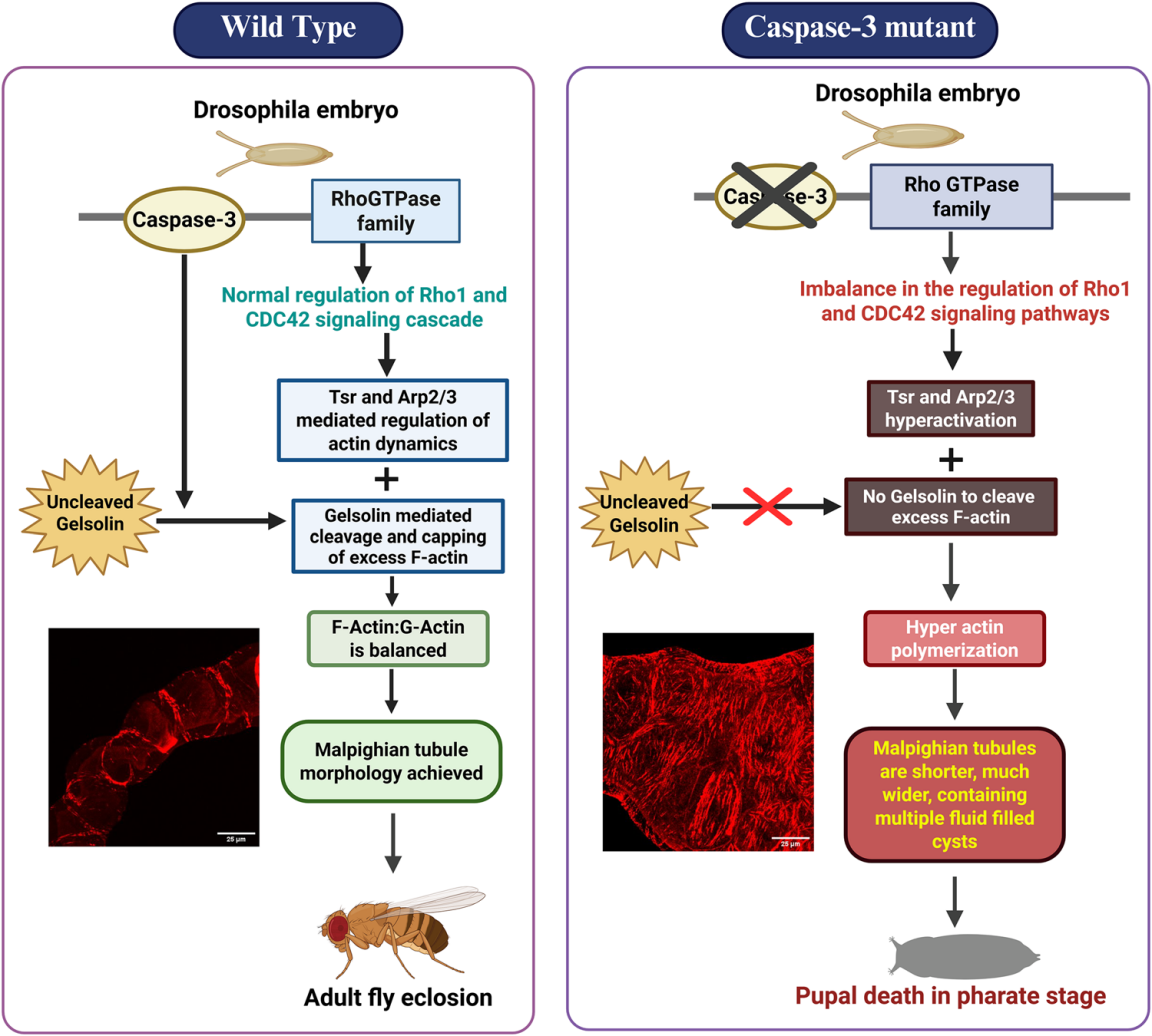
Work Summary. Drice/Caspase 3 regulates Rok, Gelsolin and CDC42 levels to maintain the cytoskeletal dynamics of the MTs.

In Malpighian tubules of *Drosophila melanogaster*, Drice/Caspase 3, directly or indirectly, regulates Rok, Gelsolin and CDC42 levels to maintain the dynamic balance of F- and G-actin. In Drice mutants, Rok activation fails, and Gelsolin remains inactive, while CDC42–Arp2/3 compensates with excessive cortical actin polymerization. This imbalance leads to hyper-dense filaments, polarity defects, and abnormal tubule morphology. As represented in Fig. 6.

These findings highlight caspases as non-apoptotic regulators of cytoskeletal remodeling and suggest broader implications in other tissues where actin homeostasis and Rho GTPases are critical. Future work could focus on defining how Drice influences Rho1 nucleotide cycling and whether similar caspase–Rho–actin pathways operate in vertebrate systems.

## Material and methods

### *Drosophila* stock husbandry

*Drosophila* stocks were raised on a standard cornmeal fly media containing 0.046% w/v cornmeal, 0.045% w/v sucrose, 0.018% w/v yeast powder, 0.007% w/v agar powder, with an additional 0.003% v/v propionic acid and 0.003% w/v hydroxybenzoic acid methyl ester. The stocks were kept in a controlled environment with a room temperature of 25 ± 2 °C, and experimental flies were reared in BODs. Genetic crosses based on the Gal4/UAS system were maintained at 29°C under a humidity-controlled 12-h light/12-h dark cycle. The *Drosophila* used for experiments are: Oregon R^*+*^*, Drice*^*∆2C8*^ (a null deletion mutation of fly line kind gift from Dr. Masayuki Miura lab)*, UAS-DriceRNAi (*kind gift from Dr. Masayuki Miura lab*), UAS RhoGEF2 (BL-9387), UAS Rok 2B1(BL-6670), UAS-Rok RNAi (BL-28797), UAS-mDia RNAi (BL-35479), UAS-Arp14d RNAi (BL-27705), UAS-Arp3CA RNAi (4560R-3), UAS-Gelsolin RNAi (BL31205), c42-Gal4, UO-Gal4 (kind gift from Dr. J.A.T. Dow*.

### Sample size selection

The MTs of 118-120h old, wandering third instar larvae were taken for most of the experiments. Experiments were not sex specific; post-eclosion, three-day-old flies were used for the genetic cross setup. Min 20 larvae were selected for each experiment, and all the experiments were repeated at least three times.

### Immunostaining

The MTs were dissected from the wandering third instar larvae in 1xPBS (phosphate buffer saline). Immediately after dissection, tissues were transferred in 4% PFA (paraformaldehyde) fixative for 45 min at room temperature. After fixation, the tissue was washed in 0.5% PBST (wash buffer) (0.5% Triton X-100 in 1xPBS). Following fixation, tissues were incubated in blocking solution (0.1% Triton X-100, 0.1% BSA, 10% FBS, 0.1% deoxycholate, 0.02% thiomersal) for 2 h at room temperature and then incubated in primary antibody overnight. Samples were washed thrice in wash buffer afterwards, followed by incubation in blocking solution again for 2 h at RT and incubated in the secondary antibody for 2 h at RT. After incubation in secondary antibody, tissues were washed again in 0.5% PBST and counterstained using DAPI (4′,6-Diamidino-2-Phenylindole, Dihydrochloride, Thermo-Fisher Scientific, Cat# D1306) (1 μg/mL) to visualize the nuclei of tissues. Samples were then washed three times and finally mounted in mounting media (DABCO) on a glass slide.

Blocking solution was used as dilutant for the antibodies.

**The following primary antibodies were used for immunohistochemistry:**

anti-Rho1 antibody raised in mouse (1:20, p1D9, DSHB),

anti-Rho1 antibody raised in rabbit (1:800, 10749-1-AP, Protein tech),

anti-Rho1 antibody raised in mouse (1:300, SC0418, Santa Cruze),

anti-CDC42 antibody raised in rabbit (1:100, 10155-1-AP, Protein tech),

anti-Rok antibody raised in rabbit (1:200, Gly1114, CST),

anti-Gelsolin antibody raised in rabbit (1:200, D9W8Y, CST),

anti-dlg antibody raised in mouse (1:20, 4F3, DSHB).

**The secondary antibodies used for the immunohistochemistry are as follows**:

donkey anti-mouse AF 555 (A31570),

goat anti-mouse AF 647 (A21050),

donkey anti-rabbit AF 555 (A31572) and

goat anti-rabbit AF 647 (A32733) from Invitrogen.

### Phalloidin staining

MTs from 118 to 120 h old wondering stage larvae were dissected in chilled 1xPBS and fixed in 4% PFA for 45 min at RT. Tissues were washed thrice in wash buffer (0.5% PBST in 1xPBS) and incubated in phalloidin atto 550 (Sigma) for 2 h. Tissues were washed again thrice in washing buffer and counterstained with DAPI for 20 min at RT. Tissues were washed and mounted in mounting media (DABCO) on a glass slide and visualized under the microscope.

### Western blotting

Third instar *Drosophila* larvae, aged 119–120 h post-hatching, were dissected, and MTs were collected for protein extraction. MTs were isolated in chilled 1X PBS, then transferred to cold RIPA buffer containing 100 mM Tris-Cl (pH 6.8), 100 mM NaCl, NP-40, EDTA, NaF, NaO₂V₄, 20% glycerol, a protease inhibitor cocktail, and 2 mM PMSF. Next, the tissues were homogenized and centrifuged at 12,000 RPM for 20 min at 4 °C, and the resulting supernatant was collected for its protein content. Protein was quantified using Bradford’s assay, and 30 µg of protein was prepared with sample buffer (100 mM Tris-Cl, pH 6.8, 4% SDS, 0.2% bromophenol blue, 20% glycerol, 100 mM DTT, and 2 mM PMSF) in a 1:1 ratio. The samples were boiled for 5 min and subjected to SDS-PAGE. Proteins were electro-transferred onto a PVDF membrane at 100 V for 1.5 h at 4 °C using a wet transfer apparatus. Following transfer, membranes were blocked in 4% BSA dissolved in 0.1% TBST for 2 h, then incubated overnight at 4 °C with the primary antibody. Membranes were washed in 0.1% TBST at RT for 15 min each washing. They were then incubated with HRP-conjugated secondary antibody for 2 h at room temperature, followed by another three TBST washes. Signal detection was performed using a ChemiDoc system (Amersham AI680) with ECL substrate (Bio-Rad). All antibodies were diluted in 3% blocking buffer.

**The following primary antibodies were used for Western Blotting:**

anti-Rho1 antibody raised in rabbit (1:1500, 10749-1-AP, Protein tech),

anti-Rho1 antibody raised in mouse (1:1000, SC-0418, Santa Cruze),

anti-CDC42 antibody raised in rabbit (1:1000, 10155-1-AP, Protein tech),

anti-Rok antibody raised in rabbit (1:1000, Gly1114, CST),

anti-Gelsolin antibody raised in rabbit (1:1000, D9W8Y, CST),

anti-actinin antibody raised in rabbit (1:1000, 2G3-3D7, DSHB).

**The secondary antibodies used for Western blotting are as follows**:

HRP tagged anti-mouse IgG (1:1500, Invitrogen)

HRP tagged anti-mouse IgG (1:1500, Invitrogen).

### F-actin and G-actin cellular fractionation

MTs from 119–120 h AEL larvae were dissected in F-actin stabilization buffer (50 mM PIPES, pH 6.9; 50 mM KCl; 5 mM MgCl₂; 5 mM EGTA; 1 mM ATP; and 0.5% NP-40 in Milli-Q water) at 37 °C, supplemented with protease inhibitor as per the manufacturer’s instructions (SigmaFAST™ Protease Inhibitor Tablets, Cat. No. S8820). The dissected MTs were mechanically homogenized at 37 °C, and the homogenate was centrifuged at 4000 × *g* to remove undissolved cellular debris. The resulting supernatant was transferred to fresh labeled microcentrifuge tubes (MCTs). The cleared supernatant was then centrifuged at 100,000 × *g* at 37 °C to precipitate the F-actin fraction. Following centrifugation, the G-actin–containing supernatant was transferred to a separate labeled MCT, while the F-actin pellet was resuspended in F-actin depolymerizing/G-actin buffer (10 mM Tris-Cl, pH 7.5; 0.5 mM EGTA; 0.2 mM ATP; 1 mM DTT; 0.1% SDS in water) using a volume equal to that of the stabilization buffer used initially. The F-actin pellet was incubated in this buffer to allow its steady conversion into soluble G-actin.

Equal volumes of the F-actin and G-actin fractions from each experimental group were then subjected to western blotting. *Note:* Since the F-actin fraction is largely devoid of other cellular proteins, loading control antibodies cannot be used for this fraction. Therefore, equal loading by volume is essential for a valid comparison. As this experiment provides a relative assessment, the F-actin and G-actin fractions can be compared across samples. However, when a suitable endogenous control antibody is available (e.g., for the G-actin fraction), this method allows accurate quantification of the relative abundance of F-actin and G-actin.

### Immunoprecipitation and immunoblotting

Third instar larvae of *Drosophila*, 119–120 h after hatching, were dissected to obtain MTs for protein extraction. The MTs were washed in chilled 1X PBS and then moved to cold RIPA buffer containing 100 mM Tris-Cl (pH 6.8), 100 mM NaCl, NP-40, EDTA, NaF, NaO₂V₄, 20% glycerol, a protease inhibitor cocktail, and 2 mM PMSF. For IP 20 µl of the Protein G Plus-Agarose Suspension beads (IP04, Milipore) was taken and washed thrice in 1xPBS at 4 °C. After washing, beads were incubated with the desired primary antibody (5–10 µg in 500 µl 1xPBS) for 1 h at RT. Following the incubation, the antibody was restored, and the bead-antibody was washed thrice in 1xPBS at 4 °C. Antibody-incubated beads were then added in 1–2 mg protein and left for 2 h at 4 °C. These beads were again washed 3–5 times in 1xPBS and subjected to immunoblotting or mass-spectroscopy based upon the desired outcome.

**The following antibodies were used for IP:**

rabbit anti-Rho1 antibody (1:1500, 10749-1-AP, Protein tech),

mouse anti-Rho1 antibody (1:1000, SC0418, Santa Cruze),

rabbit anti-Gelsolin antibody (1:1000, D9W8Y, CST).

### RNA isolation and cDNA preparation

MTs from 118 ± 1 h old post eclosion larvae were collected and immediately processed in TRI reagent (T9424, Sigma Aldrich) and stored at −80 °C until extraction. Total RNA was isolated using the TRIzol method, following the manufacturer’s protocol (Sigma-Aldrich, India), including DNase I (Thermo Fisher Scientific, Cat# 89836) digestion step to remove genomic DNA. RNA concentration and purity were measured spectrophotometrically (A260/A280 and A260/A230 ratios), and integrity was assessed by electrophoresis. First-strand cDNA was synthesized from 200–500 ng total RNA in 20 µL reactions using a reverse transcriptase with a mixture of random hexamers and oligo(dT) primers, following the enzyme supplier’s protocol. Reactions were incubated at 25 °C for 10 min (primer annealing), 42–50 °C for 30–60 min (extension), and heat-inactivated at 70 °C for 10 min. cDNA was diluted 1:5–1:20 in nuclease-free water for qRT-PCR; identical treatment was applied to RT control reactions.

### qRT-PCR

Quantitative PCR was performed using a commercial 2× qRT-PCR master mix (SYBR Green) in optical 96-well plates. Reaction composition for a 10 µL SYBR Green (SYBR Green, Genetix, Cat# PKG025-A) reaction was: 1× master mix, 200–400 nM each primer, and 1–2 µL diluted cDNA (final volume 10 µL). Plates were sealed, briefly centrifuged, and run on a real-time PCR instrument. Typical cycling conditions were: 95 °C for 2–3 min (activation), followed by 40 cycles of 95 °C for 10 s and 60 °C for 20–30 s with fluorescence acquisition at the annealing/extension step. A melt-curve was acquired for SYBR assays (65–95 °C, incremental steps). No-template controls (NTC) and −RT controls were included for every primer pair. Each biological condition included at least three independent biological replicates. Technical replicates (duplicate or triplicate wells) were measured for each sample-gene combination. Reference gene stability was assessed across all samples and conditions; RP49 and GAPDH were used as reference genes, and their geometric mean was used for normalization. Wells with atypical amplification curves, high replicate variance (>0.5–0.7 Ct), or NTC amplification were inspected and excluded when justified. Primers used: the following primers were used for qRT-PCR

***RP49*** Forward:5′- TTGAGAACGCAGGCGACCGT-3′

Reverse:5′- CGTCTCCTCCAAGAAGCGCAAG-3′

***Rho1*** Forward: 5´-ATTCCTGAAAAATGGACCCC-3´

Reverse:5´-GTTGGGATCATTTCGCAAAT-3´

***CDC42*** Forward: 5´-CTTCCTTGTCTGCTTTTCGG-3´

Reverse: 5´-AATGGTGTGTAATCTCGGGC-3´

***RAC***Forward: 5´-GGAAATCGAACCATGCAGGC-3´

Reverse: 5´-GTCGAACACGGTGGGTATGT-3´

***Rok***Forward: 5´-ACCGATGGCAGCAAGATATC-3´

Reverse: 5´-GACATTAACCGCATACGGCT-3´

***LIMK***Forward: 5´-CCAGCCCAAGTACTCCCTTG-3´

Reverse: 5´-GATCTTCACGGACCAGGCAA-3´

***Tsr***Forward: 5´-GCTCTCAAGAAGTCGCTCGT-3´

Reverse: 5´-GCAATGCACAGTGCTCGTAC-3´

***Arp2***Forward: 5´-CAAGGGTCGAAATGTTATCGTC-3´T

Reverse: 5´-AATGTGGGTGGGGAAATTGCT-3´

***Arp3***Forward: 5´-GAGGGCTATGTGATCGGCTC-3´

Reverse: 5´-TAGCAGTCTCGAGGCTCTGT-3´

**Gelsolin** Forward: 5´- GAAACACGACTATTCCAGGTCAA-3´

Reverse: 5´- TGATAGCCTTTAGCTTCTCAACG-3´

**Drice** Forward: 5´-TGTCGGCCCACCCTTATCTA-3´

Reverse: 5´-TGGACGACCATGACACACAG-3´

### Microscopy and image analysis

All the images were scanned using a Zeiss LSM-900 confocal microscope using Zen software (version 3.4). For imaging, 20× objective and 40× objectives were used on 1× zoom and 63× objective on 1.5× zoom were used, with 2.5 µm optical section interval. The optimum setting was used for image acquisition on different days. Average fluorescence per unit pixel was taken into consideration for intensity-based quantification rather than overall intensity, to normalize the size difference of the tubule area, especially considering the highly widened tubule of Drice mutants. Scanned images were processed using ImageJ software (NIH, USA) (available at ImageJ.nih.gov/ij) and Adobe Photoshop 2021 (version 22.4.2) was used to create image panels.

### F-actin thickness measurement

Super-resolution of F-actin strands was performed using the NanoJ-SRRF plugin in ImageJ. Filament thickness was then quantified by manual measurements on maximum-intensity projection images derived from Z-stack acquisitions.

### Fourier-based directionality analysis

This analysis was performed to quantify actin filament organization in Malpighian tubules. Acquired images were first converted to grayscale, and maximum-intensity Z-projections of the F-actin channel were generated. The resulting projection images were then analyzed using the Directionality plugin in ImageJ to assess actin filament orientation.

### Statistical analysis

All the experiments were repeated at least three times, and each time, a minimum of 10 tissues were taken for the experiment. All the statistical analysis was performed using GraphPad Prism 8.4.2. Unpaired Student’s *t* tests with Bonferroni correction for multiple comparisons for fold change with only two groups, Two-way ANOVA, followed by Tukey’s post hoc multiple-comparison test for qRT-PCR with more than two groups and Welch’s one-way ANOVA test was performed for F-/G-actin ratio comparison and Fourier-based directionality analysis. *p*-value ≤ 0.05 was taken as a significant difference, and the degree of significance was as follows: *p*-values ≤ 0.05, *p* ≤ 0.01, *p* ≤ 0.001, and *p* ≤ 0.0001 are signifying*, **, ***, and ****, respectively; with *p*-value > 0.05 representing a non-significant statistical difference.

## Supplementary information

Supplementary word file

Supplementary Table 1

## Data Availability

The data supporting the findings of this study are available within the article and its Supplementary Information files. Additional data are available from the corresponding author upon request.
