# Supplementary material for: Caspase-3/Drice as a critical regulator of actin dynamics through its dual control of small RhoGTPase family and Gelsolin in the Malpighian tubules of Drosophila
Source: Cell Death Discov. 2026 Apr 1;12:214. doi: 10.1038/s41420-026-03061-7 (PMC13168378; doi:10.1038/s41420-026-03061-7)
Supplement: Supplementary file 1 — Supplementary word file [file 41420_2026_3061_MOESM1_ESM.docx]

*
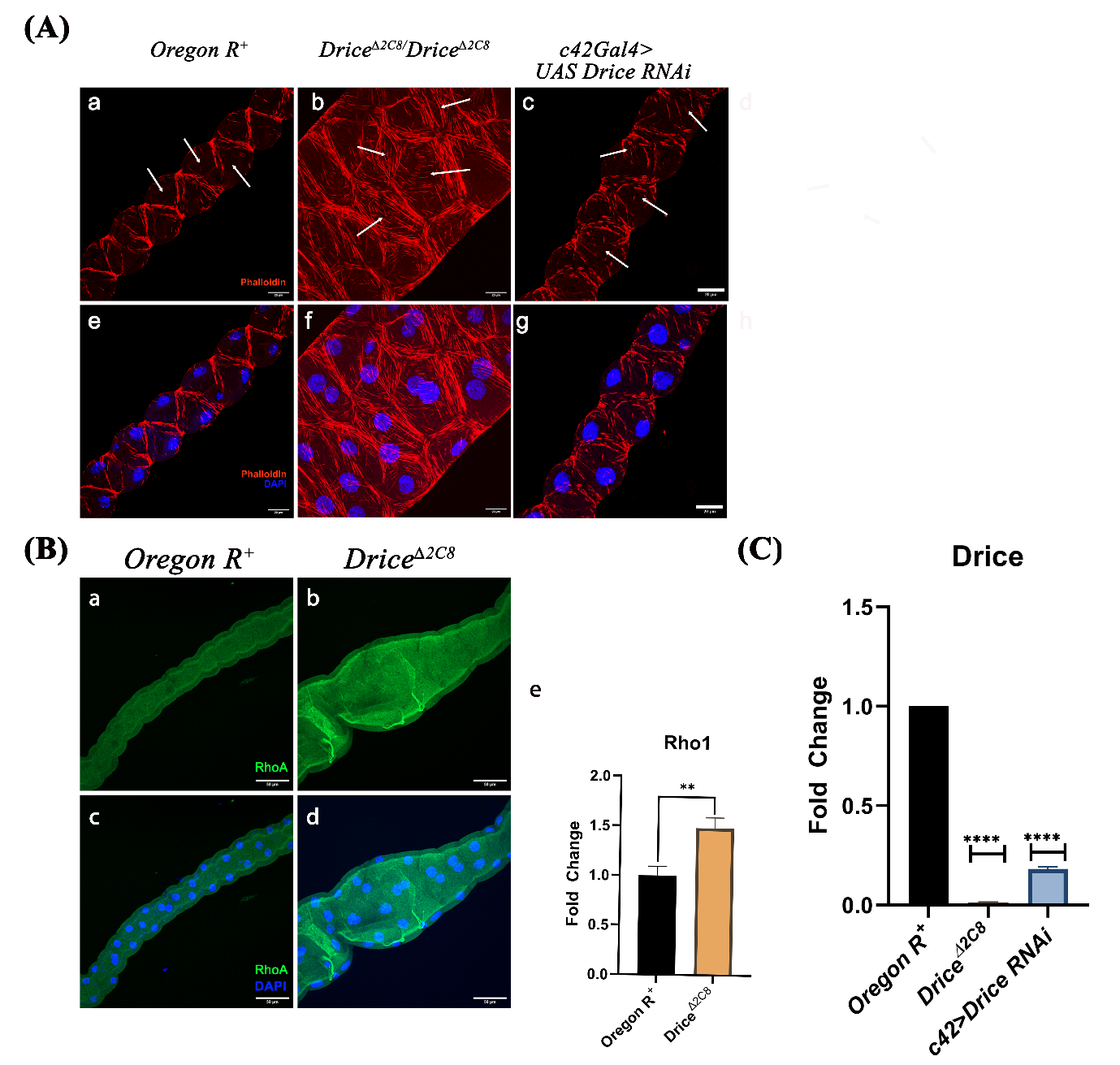
*

**Figure S1: Status of F-actin and Rho1 protein in Drice knockdown. (A) Confocal projection image of MTs of 3^rd^ instar wandering stage larvae showing the F-actin organization**. In Drice mutants (A-b, f) and Drice RNAi (A-c, g) F-actin shows marked disorganization with abundant cytosolic actin fibers compared to the wild type (A- a & e). Scale bar is 20 µm. **(B) Z-project of confocal scanning showing the expression of Rho1 protein in the MTs of 3^rd^ instar larvae**. Rho1 protein expression levels are highly elevated in Drice^Δ2C8^/Drice^Δ2C8^ larvae (B-b & d) as compared to the wild type (B-a & c). Scale bar is 50 µm. Statistical significance was determined using a two-tailed Mann–Whitney U test. p-value ≤ 0.05 is considered significant, with reference *p < 0.05, ** p <0.01, ***p < 0.001, and ****p < 0.0001. Bar graphs are showing Mean ± SEM value. Data are presented as median with interquartile range from n = 3 independent biological replicates. **(C)** **Transcript levels of the Drice** in the MTs of 3^rd^ instar Drice^Δ2C8^/Drice^Δ2C8^ and c42 GAL4>UAS Drice RNAi larvae when compared to the Oregon R^+^ in qRT-PCR (RP49 was used as internal control). Statistical significance was determined using a two-tailed Mann–Whitney U test. p-value ≤ 0.05 is considered significant, with reference *p < 0.05, ** p <0.01, ***p < 0.001, and ****p < 0.0001. Bar graphs are showing Mean ± SEM value. Data are presented as median with interquartile range from n = 3 independent biological replicates.

*
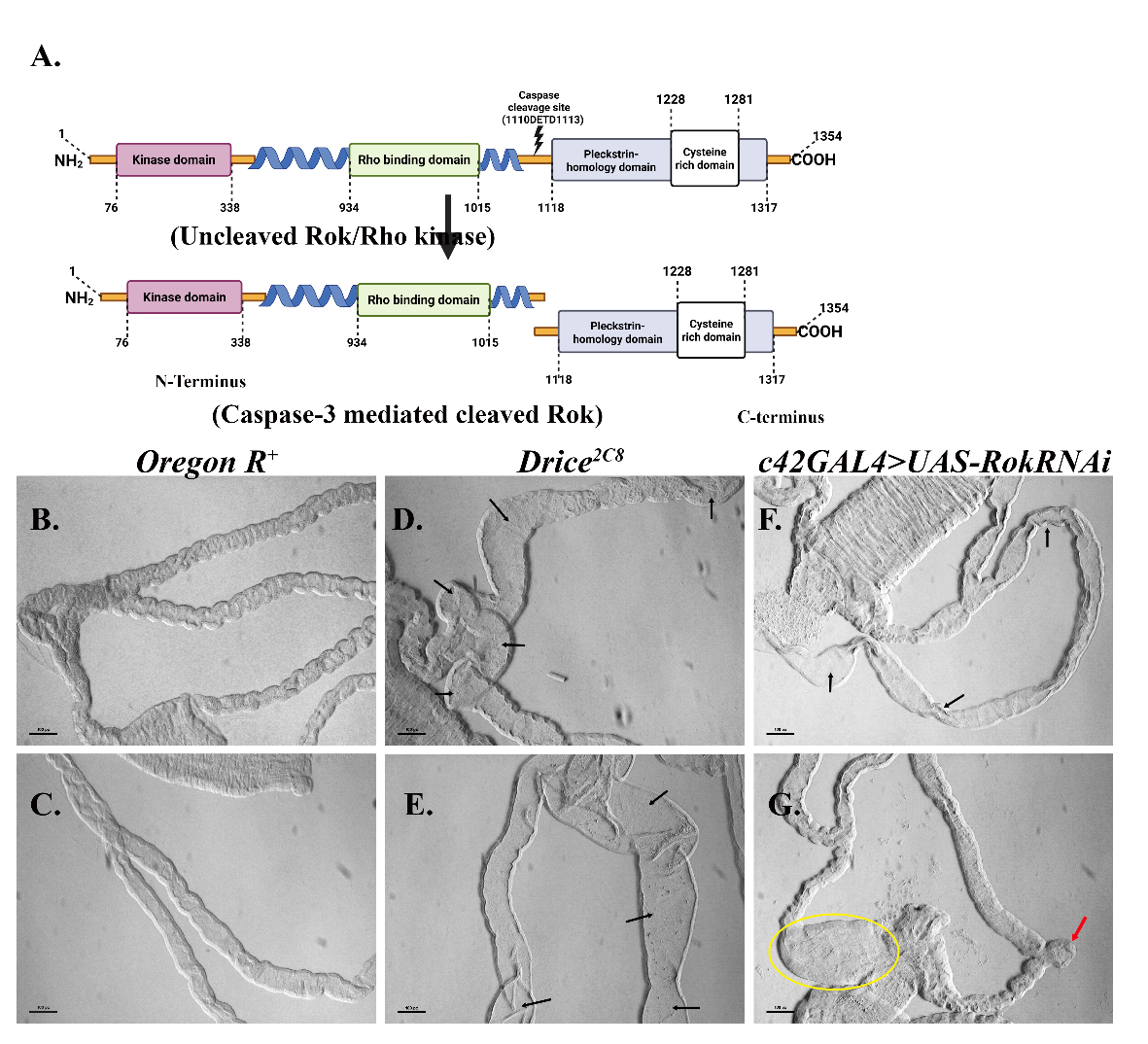
*

***Supplementary Figure S2: Caspase-3 mediated cleavage activation of Rok protein and morphology of the MTs in various genetic backgrounds. A.*** *Caspase-3 mediated cleavage of the Rok protein, rendering Rok in constitute active form.* ***B-C,*** *Normal tubule morphology in wild type,* ***D-E,*** *cystic tubules with multiple fluid filled cysts in Drice mutants. Black arrowheads are showing the cysts.* ***F-G,*** *tubule morphology in Rok knockdown. Furthermore, Rok knockdown (c42-Gal4>UAS-RokRNAi) show severe morphological defects of MTs (Fig. 2-F, G). These included multiple fluid-filled cysts (black arrows in Fig. 2-F), swollen balloon-like ureters (yellow circle in Fig. 2-G), and, in some cases, branching of the tubules (red arrow in Fig. 2-G). Scale bar is 100 µm.*

*
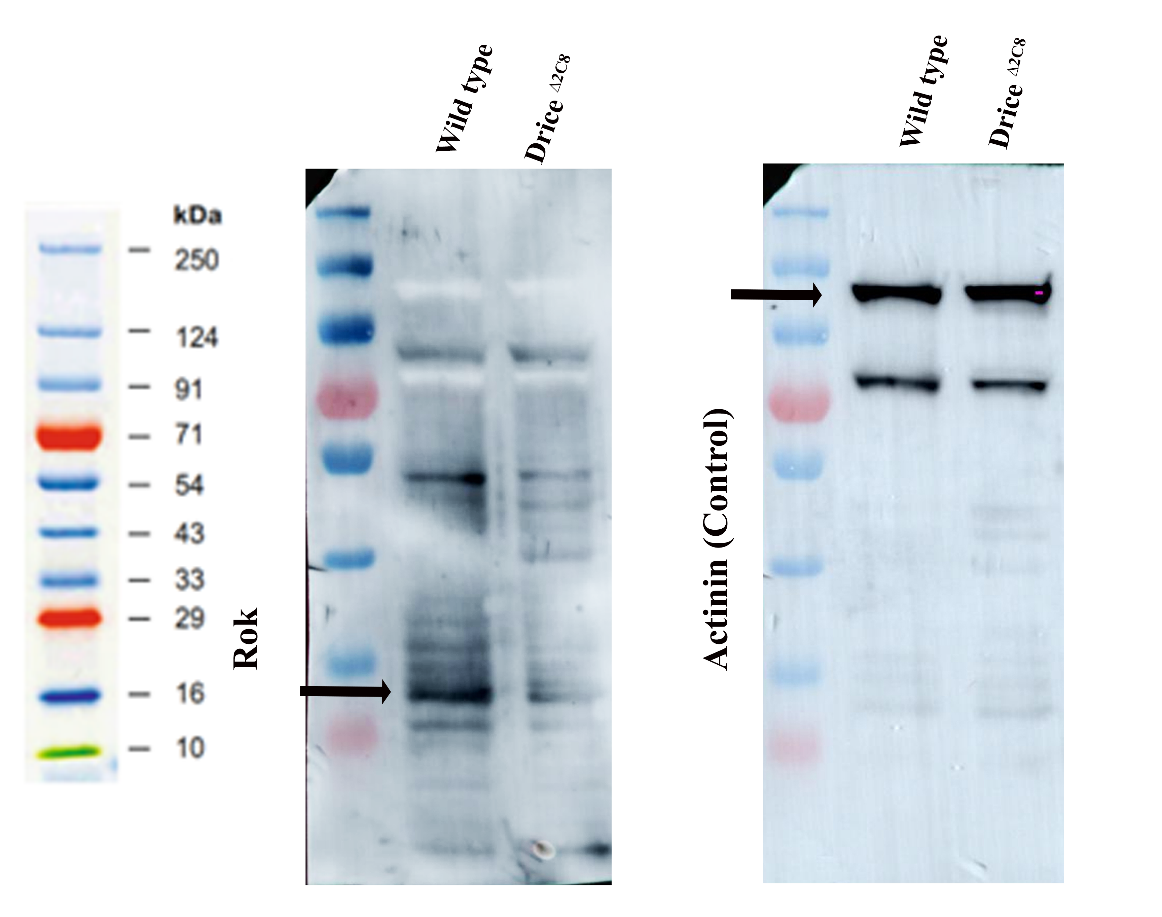
*

***Supplementary Figure S3: Whole western blot using anti-Rok (Rho kinase) antibody:*** *This image is showing the whole blot using Caspase mediated C-terminal cleaved Rok specific anti-ROK1 antibody, of ~30kDa size band. Actinin (~100 kDa) was taken as endogenous control (as actin levels were variable in control (as indicated by immunostaining data) and experimental ground that is why actinin protein, which is also highly conserved structural protein was taken as endogenous control). Black arrowheads are showing the region shown in the manuscript.*

*
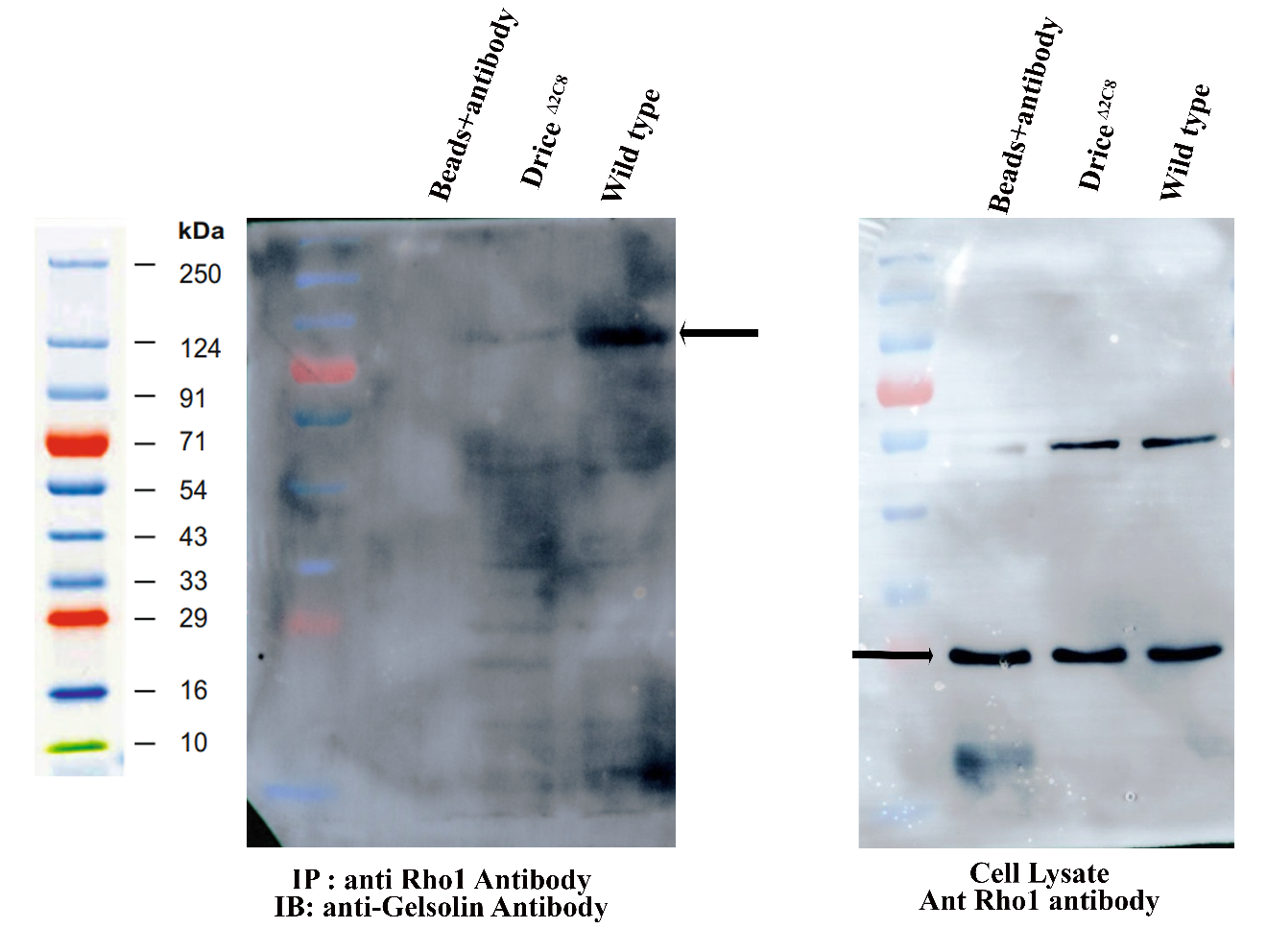
*

***Figure S4: Whole blot of the co-IP using anti-Rho1 antibody:***

*Immunoprecipitation was done using anti-Rho1 antibody and ~1.5 mg protein samples. The antibody pooled sample was subsequently subjected to the immunoblotting using anti-gelsolin antibody to check the protein-protein interaction between the Rho protein and Gelsolin protein. Cell lysate was later subjected to blotting showing the presence of the Rho1 protein in the cell lysate, beads + protein sample was taken as negative control. Black arrowheads are showing the region shown in the manuscript.*

*
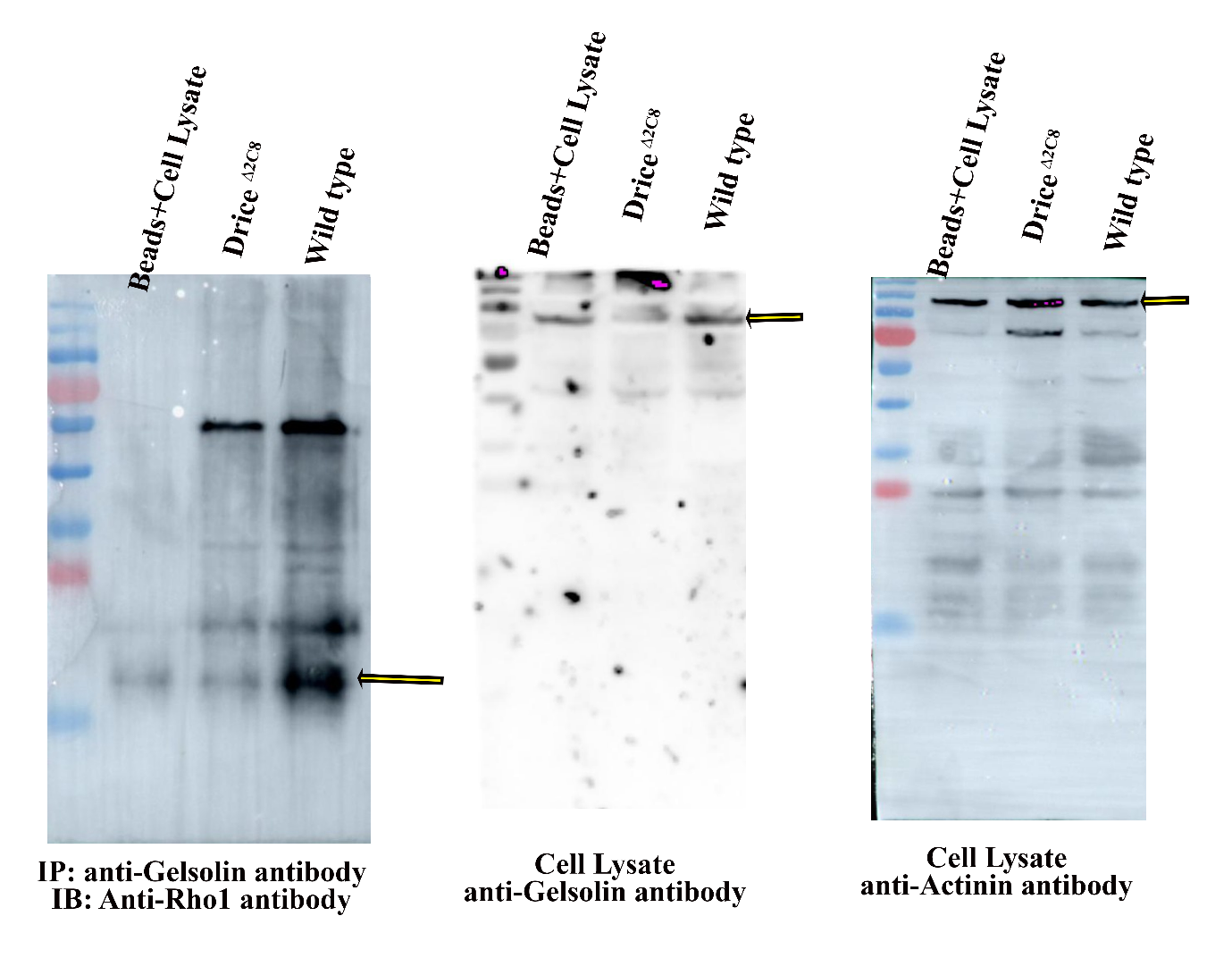
*

***Supplementary Figure S5.*** ***Whole blot of the co-IP using anti-Gelsolin antibody for pool down:***

*IP and IB condition were reversed in this experiment to cross check the reactivity of Rho1 and Gelsolin proteins. Immunoprecipitation was done using anti-Gelsolin antibody and ~1.5 mg protein samples. The antibody pooled sample was subjected to the immunoblotting using anti-Rho1 antibody to check the protein-protein interaction between the Gelsolin and Rho1 proteins. Cell lysate was later subjected to blotting showing the presence of the Gelsolin protein in the cell lysate, beads + protein sample was taken as negative control. Black arrowheads are showing the region shown in the manuscript.*

*
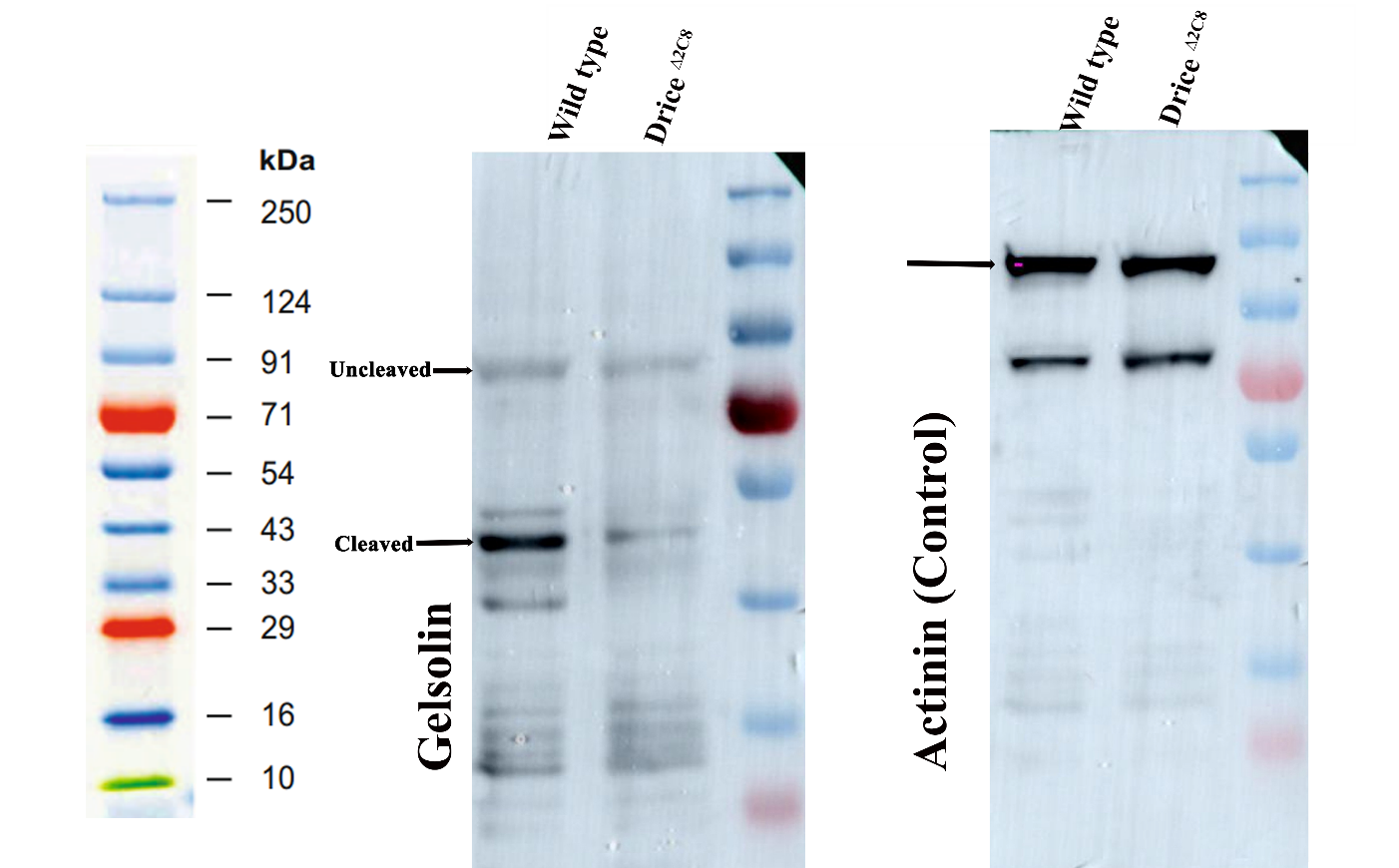
*

***Supplementary Figure S6.*** ***Whole western blot using anti-Gelsolin antibody:*** *Western blot using anti-Gelsolin antibody was performed, marking both uncleaved (~80 kDa) and cleaved (Caspase mediated cleavage ~48 kDa) band size. Actinin (~100 kDa) was taken as internal control. Black arrowheads are showing the region shown in the manuscript.*

*
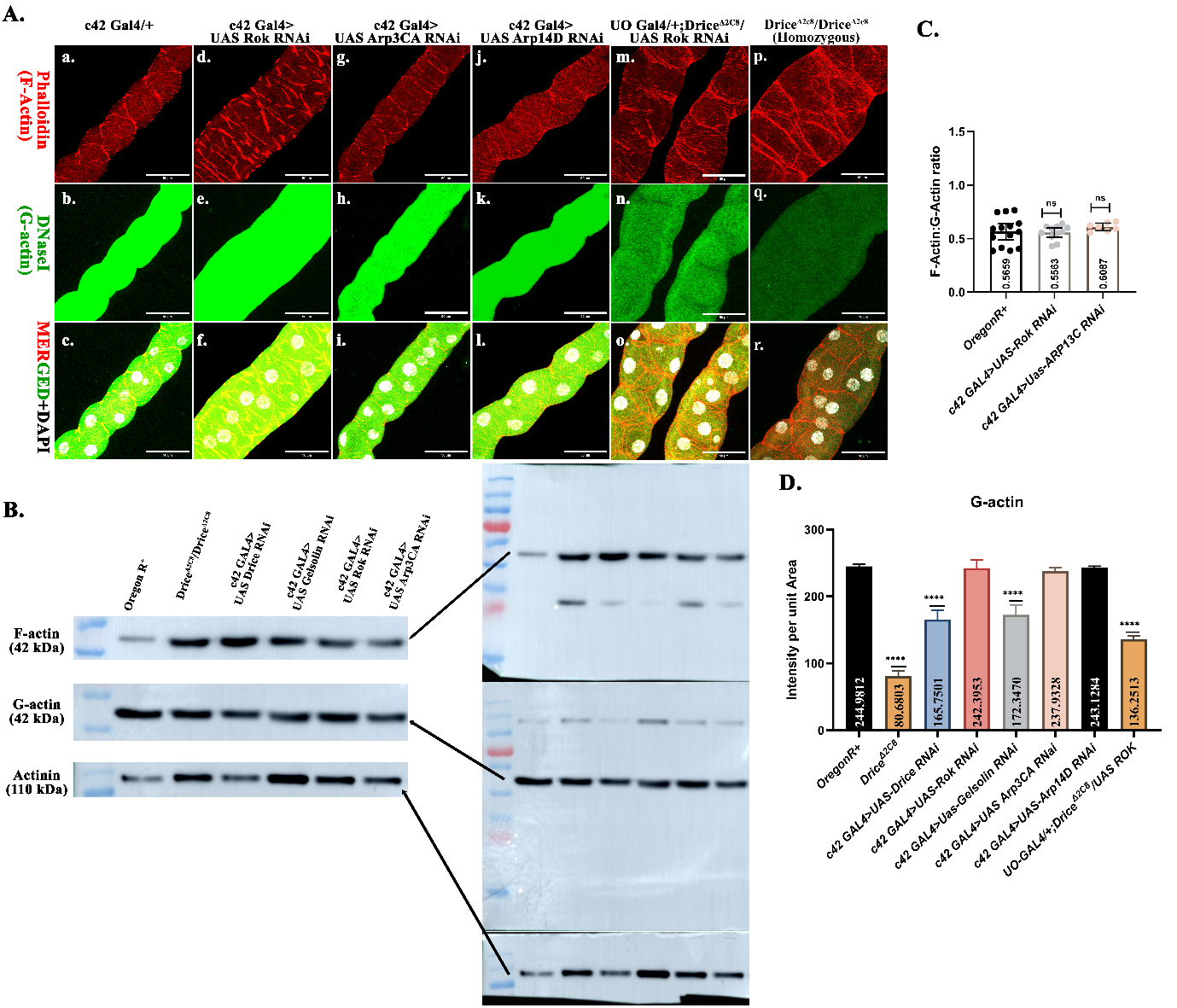
*

***Supplementary Figure S7: Status of F-actin, G-actin and F-actin:G-actin ratio in UAS-Rok RNAi, UAS Arp3CA RNAi, UAS rp14D RNAi, UO-GAL4/+; Drice^∆2C8^/UAS Rok RNAi background:*** ***(A) F-actin organization and G-actin levels in various genetic background:*** *Rok-RNAi (A, d-f), Arp3CA RNAi (A, g-i), Arp14D RNAi (A, j-l), show disrupted F-actin organization as reported earlier, however, they does not show any change in G-actin levels; G-actin level remain similar to the wild type and c42 GAL4 driver alone (A, a-c). In UO-GAL4/+; Drice^∆2C8^/UAS Rok RNAi (A, m-o) genetic background G-actin levels are better than in Drice mutants alone. Whereas, fig A, p-r is showing Drice mutants in homozygous condition. Magnitude of Scale bar is 50 µm.* ***(B) Western blot showing the G-actin and F-actin levels in above mentioned genetic backgrounds. (C) F-actin:G-actin ratio in various genetic background:*** *F-actin:G-actin ratio remains unaltered in Rok-RNAi & Arp-RNAi genetic background.* ***(D) G-actin levels in various genetic background:*** *Drice knockdown and Gelsolin knockdown reduces G-actin levels significantly. However, Arp and Rok knockdown does not show reduction in the endogenous G-actin levels. UO-GAL4/+; Drice∆2C8/UAS Rok RNAi increased the G-acti levels when compared to the Drice mutants alone. Welch’s one way ANOVA test with Tukey’s multiple comparison test was done to determine the statistical significance. p-value < 0.05 is considered significant, with reference *p < 0.05, ** p <0.01, ***p < 0.001, and ****p < 0.0001. Bar graphs are showing Mean ± SEM value. n = 5 (technical replicate) * 3 independent biological replicates.*

**
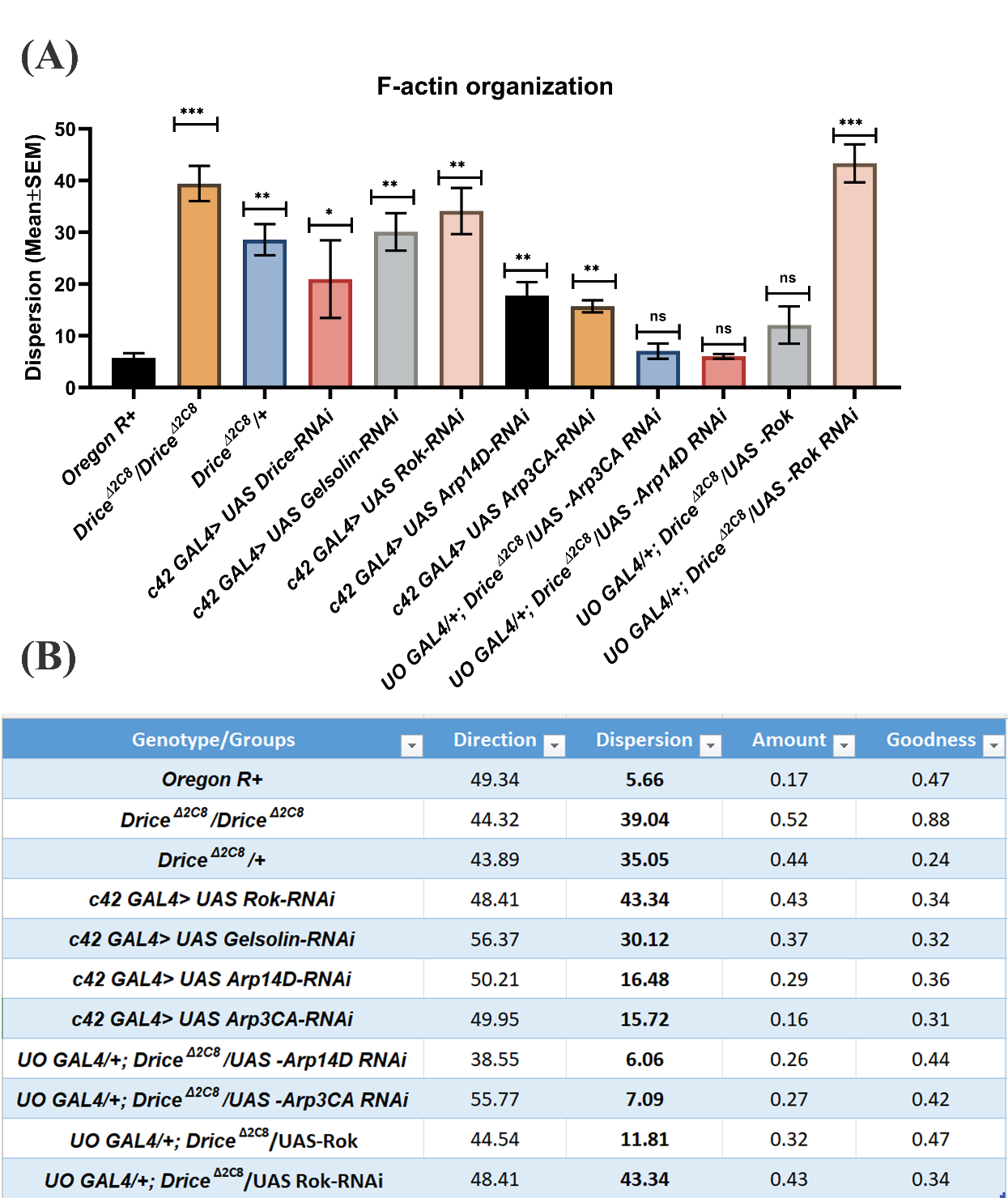
**

***Supplementary Figure S8: F-actin organization across various genotype. (A)*** *Average dispersion angle of the F-actin in different genetic background.* ***(B)*** *Fourier-based directionality analysis of phalloidin-stained Malpighian tubules was performed using the Directionality plugin in Fiji/ImageJ. The table summarizes the dominant orientation (Direction), angular dispersion (Dispersion), fraction of pixels contributing to the dominant orientation (Amount), and goodness of fit (Goodness) for each genotype. Angular dispersion was used as the primary quantitative measure of actin organization, with lower dispersion values indicating higher filament alignment and higher values indicating actin disorganization. Direction values are shown for descriptive purposes only. Measurements were obtained from identical regions of interest along the tubule epithelium, and values represent pooled measurements across analyzed tubules for each genotype.*
